# Supplementary material for: Deconstructing isolation-by-distance: The genomic consequences of limited dispersal
Source: PLoS Genet. 2017 Aug 3;13(8):e1006911. doi: 10.1371/journal.pgen.1006911 (PMC5542401; doi:10.1371/journal.pgen.1006911)
Supplement: S1 Text — (PDF) [file pgen.1006911.s006.pdf]

S1 Text: Identity-by-descent estimation for Z-linked markers

We used a method-of-moments approach similar to that described in [1] to estimate the proportion of the genome shared identical-by-descent for the Z chromosome. We used PLINK to calculate population allele frequencies for each SNP and to count the number of SNPs shared identical-by-state (IBS) for each pair of individuals. Due to the inheritance mode of the Z chromosome, identity-by-descent (IBD) calculations depend on the sex of the individuals in a pair. In birds, males (ZZ) have two copies of the Z and females (ZW) have one. Male-male comparisons of the Z chromosome are equivalent to autosomal comparisons, and the approach is fully described in [1]. Therefore we only describe male-female and female-female comparisons here. We use the same notation as Purcell *et. al.* 2007 [1] to ease comparison. If I denotes the IBS state and Z denotes the IBD state, then we can write the prior probability of IBS sharing as:

$$P(I = i) = \sum_{z=0}^{z=i} P(I = i|Z = z)P(Z = z) \tag{1}$$

For both IBS and IBD, the three possible states are 0, 1, and 2. We can estimate $P(I|Z)$ from the allele frequencies. Let p be the frequency of the A allele and q be the frequency of the a allele. Because estimates of p and q from a finite sample are subject to ascertainment bias, we add a correction factor based on observed counts of alleles. For a single SNP, let X and Y be the number of A and a alleles in the sample, and T_z be the total number of sampled alleles. Thus $p = {}^{X}\!/_{T_z}$ and $q = {}^{Y}\!/_{T_z}$. We can then calculate $P(I|Z)$ for each SNP using this ascertainment correction as follows.

For example, for male-female pairs, $P(I = 0|Z = 0)$ is the probability of observing Z^AZ^A-Z^aW pairs or Z^aZ^a-Z^AW pairs. If we could accurately estimate p and q, then $P(I = 0|Z = 0) = p^2q + pq^2$. Given a finite sample size, there are $T_z(T_z - 1)(T_z - 2)$ possible ways of picking three Z-linked alleles (two for the male and one for the female) from a total sample of T_z alleles. Of these combinations, $X(X - 1)Y$ will be Z^AZ^A-Z^aW pairs and $Y(Y - 1)X$ will be Z^aZ^a-Z^AW pairs. Therefore, in a finite sample,

$$P(I = 0|Z = 0) = \frac{X(X-1)Y}{T_z(T_z-1)(T_z-2)} + \frac{Y(Y-1)X}{T_z(T_z-1)(T_z-2)} \tag{2}$$

We can rearrange this equation to separate out the allele frequencies and the ascertainment correction based on observed allele counts:

$$P(I = 0|Z = 0) = p^2q\left(\frac{X-1}{X}\frac{T_z}{T_z-1}\frac{T_z}{T_z-2}\right) + pq^2\left(\frac{Y-1}{Y}\frac{T_z}{T_z-1}\frac{T_z}{T_z-2}\right) \tag{3}$$

We applied this same line of reasoning to generate the full set of $P(I|Z)$.

Note that in male-female pairs and female-female pairs, $P(Z=2)=0$. For male-female pairs:

$$P(I=0|Z=0) = p^2 q \left(\frac{X-1}{X}\frac{T_z}{T_z-1}\frac{T_z}{T_z-2}\right) + pq^2 \left(\frac{Y-1}{Y}\frac{T_z}{T_z-1}\frac{T_z}{T_z-2}\right) \tag{4}$$

$$\begin{aligned} P(I=1|Z=0) = {} & p^3 \left(\frac{X-1}{X}\frac{X-2}{X}\frac{T_z}{T_z-1}\frac{T_z}{T_z-2}\right) + 2p^2 q \left(\frac{X-1}{X}\frac{T_z}{T_z-1}\frac{T_z}{T_z-2}\right) \\ & + 2pq^2 \left(\frac{Y-1}{Y}\frac{T_z}{T_z-1}\frac{T_z}{T_z-2}\right) + q^3 \left(\frac{Y-1}{Y}\frac{Y-2}{Y}\frac{T_z}{T_z-1}\frac{T_z}{T_z-2}\right) \end{aligned} \tag{5}$$

$$P(I=2|Z=0) = 0 \tag{6}$$

$$P(I=0|Z=1) = 0 \tag{7}$$

$$P(I=1|Z=1) = p^2 \left(\frac{X-1}{X}\frac{T_z}{T_z-1}\right) + 2pq \left(\frac{T_z}{T_z-1}\right) + q^2 \left(\frac{Y-1}{Y}\frac{T_z}{T_z-1}\right) \tag{8}$$

$$P(I=2|Z=1) = 0 \tag{9}$$

$$P(I=i|Z=2) = 0 \tag{10}$$

For female-female pairs:

$$P(I=0|Z=0) = 2pq \left(\frac{T_z}{T_z-1}\right) \tag{11}$$

$$P(I=1|Z=0) = p^2 \left(\frac{X-1}{X}\frac{T_z}{T_z-1}\right) + q^2 \left(\frac{Y-1}{Y}\frac{T_z}{T_z-1}\right) \tag{12}$$

$$P(I=2|Z=0) = 0 \tag{13}$$

$$P(I=0|Z=1) = 0 \tag{14}$$

$$P(I=1|Z=1) = 1 \tag{15}$$

$$P(I=2|Z=1) = 0 \tag{16}$$

$$P(I=i|Z=2) = 0 \tag{17}$$

We then sum over all L SNPs with genotype data in both individuals of a pair to obtain the expected number of SNPs with IBS state i conditional on IBD state z:

$$N(I=i|Z=z) = \sum_L P(I=i|Z=z). \tag{18}$$

We can rearrange equation 1 and substitute in the expected counts of IBS to obtain global estimates of $P(Z)$:

$$P(Z=0) = \frac{N(I=0)}{N(I=0|Z=0)} \tag{19}$$

$$P(Z=1) = 1 - P(Z=0). \tag{20}$$

Finally, we calculate the proportion of the genome shared IBD for each comparison:

$$\hat{\pi}_{MF} = P(Z = 1) \tag{21}$$
$$\hat{\pi}_{FF} = 2P(Z = 1) \tag{22}$$

The above provides an unbiased estimate of IBD for Z-linked SNPs. In [1] the IBD probabilities are transformed in order to constrain IBD estimates to biologically plausible values (*i.e.*, between 0 and 1). We skip this final transformation step here in order to avoid introducing biases when estimating IBD for the Z chromosome using a much smaller number of markers.

References

[1] Purcell S, Neale B, Todd-Brown K, Thomas L, Ferreira MA, Bender D, et al. PLINK: a tool set for whole-genome association and population-based linkage analyses. Am J Hum Genet. 2007;81(3):559–575.
