# Supplementary material for: Deconstructing isolation-by-distance: The genomic consequences of limited dispersal
Source: PLoS Genet. 2017 Aug 3;13(8):e1006911. doi: 10.1371/journal.pgen.1006911 (PMC5542401; doi:10.1371/journal.pgen.1006911)
Supplement: S2 Text — (PDF) [file pgen.1006911.s007.pdf]

S2 Text: Comparison of patterns on the autosomes and Z chromosome

Patterns of genetic diversity are expected to differ between the autosomes and the Z chromosome given the difference in inheritance patterns and sex-biased dispersal. In particular, we predict to see (1) higher levels of identity-by-descent on the Z chromosome because of its smaller effective population size, and (2) a stronger signal of isolation-by-distance because of the strong female-biased dispersal observed in the field.

We expect to see higher levels of identity-by-descent on the Z chromosome compared to the autosomes because the Z has a smaller effective population size. The effective population size of the Z is expected to be three-quarters that of the autosomes, but it can be distorted away from this value by a variety of factors, such as the effective number of male and female parents and the variance in reproductive success among individuals [1, 2]. Given the monogamous mating system of the Florida Scrub-Jay, we do not expect large sex differences in the effective number of male and female parents or variance in reproductive success. Regardless, we investigated differences in effective population size between autosomes and the Z chromosome using the pedigree, which naturally encompasses the effects of sex ratios and variance in reproductive success.

From the pedigree, we can obtain the probability that a pair of individuals is related, which we define as parent-offspring (P_p), full-siblings (P_f), or half-siblings (P_h). Using these values, we can estimate the overall probability two randomly sampled autosomal alleles coalesce in the previous generation:

$$P(\text{coal}|\text{auto}) = 0.5P_p + 0.5P_f + 0.25P_h \tag{1}$$

The probability two randomly sampled Z-linked alleles coalesce in the previous generation depends on the sex of the pair:

$$\begin{aligned} P(\text{coal}|\text{Z}) = P_{MM}[0.5P_{p,MM} + 0.75P_{f,MM} + 0.375P_{h,MM}] \\ + P_{MF}[P_{p,MF} + 0.5P_{f,MF} + 0.25P_{h,MF}] \\ + P_{FF}[P_{f,FF} + 0.5P_{h,FF}] \end{aligned} \tag{2}$$

where P_{MM}, P_{MF}, and P_{FF} are the probabilities of male-male, male-female, and female-female pairs, respectively, and $P_{p,i}$, $P_{f,i}$, and $P_{h,i}$ are the proportion of parent-offspring, full-sibling, and half-sibling pairs for each type of comparison. Assuming equal sex ratios, an assumption that is met in each distance bin, $P_{MM} = 4/9$, $P_{MF} = 4/9$, and $P_{FF} = 1/9$. We found that overall, $P(\text{coal}|\text{auto}) = 0.0032$ and $P(\text{coal}|\text{Z}) = 0.0044$. The ratio of per-generation Z to autosome coalescent probabilities is 0.72—close to the 3/4 that we expect to see in this genetically monogamous population.

To examine the geographic pattern of coalescent probabilities, we then estimated the probability that two randomly sampled alleles a distance d apart coalesce in the previous generation ($P(\text{coal}|d)$) by calculating the proportion of parent-offspring pairs ($P_p(d)$), full-siblings ($P_f(d)$), or half-siblings ($P_h(d)$) from the observed pedigree binned by geographic

distance (S9 Fig). Results for autosomal markers and Z-linked markers are plotted in S5A Fig. We found that $P(\text{coal}|\text{Z}, d)$ is greater than $P(\text{coal}|\text{auto}, d)$ only in the first four distance quantiles ($d < 2,265$ m), which makes sense given the geographic distribution of closely related pairs.

If the probability of coalescence only differs in the first four distance quantiles, what explains the higher identity-by-descent for Z-linked markers at larger distance bins? Our study population has a relatively high immigration rate, and relatedness among immigrants likely contributes to the observed patterns. Indeed, observed levels of identity-by-descent for immigrant-immigrant and immigrant-resident pairs are higher for Z-linked SNPs compared to autosomal SNPs at all but the largest distance bin (S5B Fig). Higher identity-by-descent on the Z compared to autosomes for immigrant pairs is consistent with the smaller effective population size of the Z chromosome (where here the effective population size is for lineages sampled from the pool of immigrant lineages).

To further investigate the differences between autosomal and Z-linked markers, we used spatially-explicit coalescent simulations to model expected levels of identity-by-descent as a function of geographic distance (see S3 Text for the full derivation of our simulation framework). Briefly, for a given sex-specific pair, the expected proportion of the genome shared identical-by-descent between two individuals is:

$$\hat{Z} = \sum_{g=1}^{10} \left[\prod_{k=1}^{g-1} (1 - M(s_{k,1}))(1 - M(s_{k,2})) \left[1 - P_p(d_k, s_{k,1}, s_{k,2}) - P_f(d_k, s_{k,1}, s_{k,2}) - P_h(d_k, s_{k,1}, s_{k,2})\right] \right] \times \\ [P_p(d_g, s_{g,1}, s_{g,2})\mathbb{E}(Z_p) + P_f(d_g, s_{g,1}, s_{g,2})\mathbb{E}(Z_f) + P_h(d_g, s_{g,1}, s_{g,2})\mathbb{E}(Z_h) \\ + M(s_{g,1})(1 - M(s_{g,2}))\mathbb{E}(Z_r(d_g)) + (1 - M(s_{g,1}))M(s_{g,2})\mathbb{E}(Z_r(d_g)) + M(s_{g,1})M(s_{g,2})\mathbb{E}(Z_m(d_g))] \tag{3}$$

where $s_{k,1}$ and $s_{k,2}$ are the sexes of the two individuals in a pair; $M(s)$ is the sex-specific probability that a lineage was brought into the population by an immigrant; $P_p(d_k, s_{k,1}, s_{k,2})$, $P_f(d_k, s_{k,1}, s_{k,2})$, and $P_h(d_k, s_{k,1}, s_{k,2})$ are the sex-specific probabilities a pair of individuals is parent-offspring, full-siblings, or half-siblings, respectively; $\mathbb{E}(Z_p)$, $\mathbb{E}(Z_f)$, $\mathbb{E}(Z_h)$ are the expected identity-by-descent values for parent-offspring, full-siblings, or half-siblings, respectively; and $\mathbb{E}(Z_r(d))$ and $\mathbb{E}(Z_m(d))$ are the empirical mean proportion of the genome shared identical-by-descent for immigrant-resident and immigrant-immigrant pairs conditional on distance.

We binned distances into 15 quantiles and ran 1,000 simulations of each type of comparison (male-male, male-female, and female-female) for each distance bin. Since we have nearly equal sex ratios, we then weighted the comparisons by the probability of randomly sampling two alleles from a given sex-specific pair to obtain expected overall values for autosomes.

$$\hat{Z}_{auto} = \frac{1}{4}\hat{Z}_{MM} + \frac{1}{2}\hat{Z}_{MF} + \frac{1}{4}\hat{Z}_{FF} \tag{4}$$

Simulations for Z-linked markers differ from the autosomal simulations in four ways:

1. The probability of sampling alleles from male-male, male-female, and female-female pairs is different. Expected overall identity-by-descent on the Z is:

$$\hat{Z}_Z = \frac{4}{9}\hat{Z}_{MM} + \frac{4}{9}\hat{Z}_{MF} + \frac{1}{9}\hat{Z}_{FF} \tag{5}$$

2. When simulating dispersal events back in time, females must have a male ancestor.

3. Expected identity-by-descent values for closely related individuals ($\mathbb{E}(Z_p)$, $\mathbb{E}(Z_f)$, $\mathbb{E}(Z_h)$) are sex-specific and different (see S9 Table).

4. The proportion of the genome shared identical-by-descent for immigrant-resident ($\mathbb{E}(Z_r(d))$) and immigrant-immigrant pairs ($\mathbb{E}(Z_m(d))$) is higher on the Z than on autosomes (see S5B Fig).

Note that in the analyses presented here, we use untransformed identity-by-descent values for both autosomal and Z simulations to ease comparison given the large differences in marker number.

We ran a series of simulations gradually changing the parameters from a full autosomal model to a full Z model and compared results to observed patterns for autosomal and Z-linked markers using the coefficient of determination, R^2 (see S3 Text). S6 Fig shows simulation results for three models: the full autosomal model, a model using all Z parameters except for the identity-by-descent of immigrants, and the full Z model. As expected, the full autosomal model provides a good fit to observed patterns for autosomal markers ($R^2 = 0.95$) but not Z-linked markers ($R^2 = 0.26$). Substituting in Z parameters for allele weights, dispersal, and expected identity-by-descent values for related individuals increases the expected identity-by-descent in small distance bins. This result is consistent with the observation that the probability two randomly sampled Z alleles coalesce is higher than that for two autosomal alleles at small distances only (S5A Fig). This hybrid model only slightly increases the fit for Z-linked markers ($R^2 = 0.50$) and is a poor fit for autosomal markers as well ($R^2 = 0.31$). Finally, if we incorporate identity-by-descent values for immigrants estimated from Z-linked markers, the full Z model provides a good fit for Z-linked markers ($R^2 = 0.92$) but not autosomal markers ($R^2 = -4.41$). The fact that changing identity-by-descent values for immigrants leads to a large increase in R^2 confirms that the observed Z-autosome difference is largely driven by immigration, which is not surprising given the high rate of immigration into our study population.

Another factor that influences patterns of genetic diversity on the autosomes and Z chromosome is sex-biased dispersal. Female Florida Scrub-Jays disperse significantly farther than males, which should generate stronger patterns of isolation-by-distance on the Z chromosome because males carry two copies while females carry one. We investigated this prediction by using coalescent simulations to generate predicted isolation-by-distance curves under different models of dispersal. Here, because we know that identity-by-descent values for immigrants is the major determinant of the observed differences between the Z and autosomes, we use autosomal identity-by-descent values for immigrants (middle panel of S6

Fig). Therefore, the results presented in S7 Fig are predicted means that largely ignore the contribution of immigrants to any differences between types of markers.

First, we generated predicted relationships of identity-by-descent with geographic distance for autosomal and Z-linked markers by either drawing dispersal distances from the combined empirical dispersal curve for both sexes (equal dispersal between sexes) or by drawing dispersal distances from the empirical sex-specific dispersal curve (female-biased dispersal). Results for the two different dispersal scenarios for each marker type are shown in S7A Fig. When our simulations incorporate the observed sex-bias in dispersal, the expected identity-by-descent is higher in small distance bins, and this difference is larger in models for Z-linked markers. These simulations also predict a sharper decrease in identity-by-descent with geographic distance on the Z chromosome compared to the autosomes.

We then conducted a number of simulations to assess the contribution of effective population size and sex-biased dispersal to predicted patterns of isolation-by-distance on the Z chromosome. In these simulations, we used sex-specific dispersal simulations and Z parameters, and only varied the values we used for the probability of coalescence in the previous generation, or the expected identity-by-descent values for closely related individuals ($\mathbb{E}(Z_p)$, $\mathbb{E}(Z_f)$, $\mathbb{E}(Z_h)$). Results for a full autosomal model as well as these three different Z models are shown in S7B Fig. Predicted curves differ most at the smallest distance bin. Changing the probability of sampling alleles from male-male, male-female, and female-female pairs to values for the Z chromosome but using autosomal coalescent probabilities increases identity-by-descent at the smallest distance bin to 0.038 compared to 0.031 for the full autosomal model. To disentangle the effects of smaller population size and female-biased dispersal, we ran simulations using autosomal coalescent probabilities, which are the same for all sexes, divided by $3/4$. Identity-by-descent at the smallest distance bin for this model is 0.046, indicating that the smaller effective population size of the Z chromosome does contribute to a stronger pattern of isolation-by-distance on the Z chromozome. However, female-biased dispersal also plays a role, as the model that uses sex-specific probabilities of coalescence for the Z chromosome (the full Z model) has even higher identity-by-descent at the smallest distance bin (0.049). Our simulation results indicate that, as predicted, both the smaller effective population size of the Z chromosome and female-biased dispersal cause lower effective dispersal of Z-linked markers. We do not observe stronger isolation-by-distance on the Z chromosome compared to autosomes in our empirical data because the observed patterns are largely driven by immigration, and we have fairly noisy estimates of identity-by-descent on the Z chromosome.

References

[1] Caballero A, et al. Developments in the prediction of effective population size. Heredity. 1994;73(6):657–679.

[2] Charlesworth B. Effective population size and patterns of molecular evolution and variation. Nature Reviews Genetics. 2009;10(3):195–205.
