# Supplementary material for: Deconstructing isolation-by-distance: The genomic consequences of limited dispersal
Source: PLoS Genet. 2017 Aug 3;13(8):e1006911. doi: 10.1371/journal.pgen.1006911 (PMC5542401; doi:10.1371/journal.pgen.1006911)
Supplement: S3 Text — (PDF) [file pgen.1006911.s008.pdf]

S3 Text: Derivation of coalescent model

In every population, there is a single population pedigree that describes all family relationships among individuals in the population for every generation. Gene genealogies for a given population are constrained by rules of Mendelian inheritance and the population pedigree [1]. While we know a reasonably large portion of the recent pedigree for the Archbold Florida Scrub-Jay population, we wanted to test the extent to which knowledge of dispersal and some basic statistics of the pedigree could recapitulate the observed patterns of isolation-by-distance. Therefore, we took a hybrid approach of a coalescent-style simulation, using our empirical observations about dispersal and the pedigree to generate expected levels of identity-by-descent between pairs of individuals. For simplicity, we ignore linkage, and consider identity-by-descent probabilities as referring to any single locus in the genome or proportion of the whole genome that is shared identical-by-descent. This is equivalent to assuming all loci are independent (unlinked).

Theory

We used spatially-explicit coalescent simulations similar to Malécot's models of isolation-by-distance to generate expected levels of identity-by-descent between pairs of individuals as a function of geographic distance. Malécot defined isolation-by-distance as the population genetic pattern in which genetic relatedness between a pair of individuals decreases as the geographic distance between them increases [2]. This pattern is generated in populations at equilibrium by short-distance natal dispersal. Malécot formulated his theory in terms of the coefficient of kinship, the probability that an allele randomly selected from individual I and an allele randomly selection from individual J are identical-by-descent. Let individual I born at location x be descended from parent P born at z and individual J born at w be descended from parent Q born at u. Malécot showed that the coefficient of kinship for two individuals located at x and w is a function of the mutation rate (k) and the gametic kinship chain (Fig 5B), which in turn is a function of the migration probabilities (l_{xz}), the kinship coefficient in the previous generation, and the population size (N):

$$\varphi_n(x, w) = (1-k)^2 \left[\sum_z \sum_u l_{xz} l_{wu} \varphi_{n-1}(z, u) + \sum_z l_{xz} l_{wz} \frac{1 - \varphi_{n-1}(z, z)}{2N} \right], \qquad (1)$$

where l_{xz} is the probability of migration from z to x, which is assumed to depend only on natal dispersal distance. The second summation term is the probability that the two alleles are descended from a common ancestor born at location z [2]. From previous work, we know that there is a non-negligible rate of immigration into the Archbold population [3]. We therefore extend Malécot's framework to include immigration from other outside populations. Now, two alleles from a pair of individuals either reach a common ancestor in our population or one or both lineages descend from an immigrant into the population (Fig 5C). We formulate our model in terms of the proportion of the genome shared identical-by-descent, not the kinship coefficient. We assume no mutation, which is a reasonable assumption for SNPs over

the short time-scales we are modeling. We take as input a sequence of simulated distances between a pair of lineages back over generations $(d_1,\ d_2,\ \cdots,\ d_\infty)$, where the lineages were initially d_1 distance apart. Given this sequence, we can write the expected proportion of the genome shared identical-by-descent between two individuals with such a lineage dispersal history as:

$$\hat{Z} = \sum_{g=1}^{\infty} \left[\prod_{k=1}^{g-1} (1-M)^2 [1 - P_{rel}(d_k)] \right] \times \\ [P_{rel}(d_g)\mathbb{E}(Z_{rel}) + 2M(1-M)\mathbb{E}(Z_r) + M^2\mathbb{E}(Z_m)], \quad (2)$$

where $P_{rel}(d)$ is the probability that a pair of individuals at distance d apart have a common ancestor in the next generation back, M is the probability that a lineage was brought into the population by an immigrant, and $\mathbb{E}(Z_{rel})$, $\mathbb{E}(Z_r)$, and $\mathbb{E}(Z_m)$ are the expected identity-by-descent values for related pairs, immigrant-resident pairs, and immigrant-immigrant pairs, respectively. The expression on the first line is the probability that the two individuals located at distances $(d_1,\ \cdots,\ d_{g-1})$ did not share a common ancestor or have an immigrant ancestor in the previous $g-1$ generations. The second line is the probability that two individuals either share a common ancestor or have an immigrant ancestor in generation g.

We directly estimate $P_{rel}(d)$ from the pedigree instead of using a standard Kingman coalescent model. Here, we decompose $P_{rel}(d)$ into the probability a pair of individuals is parent-offspring ($P_p(d)$), full-siblings ($P_f(d)$), or half-siblings ($P_h(d)$). Substituting these values in to obtain our final model:

$$\hat{Z} = \sum_{g=1}^{\infty} \left[\prod_{k=1}^{g-1} (1-M)^2 \left[1 - P_p(d_k) - P_f(d_k) - P_h(d_k)\right] \right] \times \\ [P_p(d_g)\mathbb{E}(Z_p) + P_f(d_g)\mathbb{E}(Z_f) + P_h(d_g)\mathbb{E}(Z_h) + 2M(1-M)\mathbb{E}(Z_r) + M^2\mathbb{E}(Z_m)], \quad (3)$$

where $\mathbb{E}(Z_p)$, $\mathbb{E}(Z_f)$, and $\mathbb{E}(Z_h)$ are the expected identity-by-descent values for parent-offspring, full-siblings, and half-siblings respectively. We then average this value over a large number of simulations of the distances between lineages. We call this model M0, as we will build more realism into it, in later sections.

Implementation

We assume that the dispersal distribution depends only on natal dispersal. The sexes of the two current-day individuals are fixed, and we randomly assign the sex of all ancestral individuals. In each generation, we simulate dispersal on a two-dimensional space as follows: we sample a dispersal distance from the empirical dispersal curve and randomly pick an angle for the dispersal movement. We then calculate the geographic distance d between the two individuals.

We obtain parameter estimates from the data. We calculate the proportion of pairs parent-offspring pairs ($P_p(d)$), full-siblings ($P_f(d)$), or half-siblings ($P_h(d)$) from the observed

pedigree binned by geographical distance (d). These values are plotted in S9 Fig. The probability an individual is an immigrant, M, is the mean proportion of adult breeders who were not born in Archbold in 2003, 2008, and 2013 (0.271). Expected autosomal and Z-linked identity-by-descent values for non-inbred pairs are listed in S9 Table. For the expected Z-linked identity-by-descent of half-siblings, we averaged that of paternal half-siblings and maternal half-siblings. Because we do not have samples from outside populations and therefore do not know the expected relatedness for immigrants, we used the empirical mean proportion of the genome shared identical-by-descent for immigrant-immigrant and immigrant-resident pairs (*i.e.*, $\mathbb{E}(Z_r)$ and $\mathbb{E}(Z_m)$). Simulations for Z-linked SNPs used untransformed identity-by-descent values. In practice, we find that the vast majority of lineages either reached a common ancestor or had an immigrant ancestor within 10 generations, so we truncated our simulations accordingly. For each type of comparison (male-male, male-female, and female-female) and each type of marker (autosomal or Z), we binned distances into 15 quantiles and ran 1,000 simulations for each distance bin. We evaluated the fit of our model using the coefficient of determination R^2:

$$R^2 = 1 - \frac{\sum_i (y_i - \hat{Z}_i)^2}{\sum_i (y_i - \bar{y}_i)^2}, \tag{4}$$

where y_i are the mean observed identity-by-descent values in distance bin i, and Z_i are the mean simulated identity-by-descent values in distance bin i.

Each set of simulations was performed twice. We first ran simulations using parameters estimated from the full dataset. One concern, however, is that using the same dataset to estimate parameters and compare model fit could result in over-fitting. Therefore, we then performed two-fold cross-validation by estimating parameters from half our data (randomly sampling half the pairs of individuals) and comparing the fit of our model to empirical estimates from the other half of the data (the other half of the pairs). We report the mean R^2 over five iterations of these cross-validation runs.

Results

The results from the cross-validation runs are similar to those obtained using the full dataset for both parameter estimation and prediction. Results from both sets of models are presented in Table 1; we discuss results from the full dataset here and in the main text. Fig 7, S10 Fig, and S11 Fig show results from the full dataset. We first used a model where all parameters were sex-averaged. This simple model (M1) explains a large proportion of the variance in observed patterns of mean proportion of the genome shared identical-by-descent as a function of distance for male-female comparisons (R^2 = 0.90), but performs less well for male-male (R^2 = 0.61) and female-female comparisons (R^2 = -0.10; Table 1). The negative R^2 value for female-female comparisons indicates that our model provided a worse fit to the data than the means. The simulations predicted a steeper decline in identity-by-descent with geographic distance than observed for female-female pairs and a shallower decline for male-male pairs (S10 Fig).

The failure of our model to reconstruct observed isolation-by-distance patterns for male-male and female-female comparisons is likely due to the fact that we used sex-averaged values for all parameters estimated from our data. Florida Scrub-Jays have different dispersal behavior between sexes, with females dispersing significantly farther than males. Therefore, we sequentially added sex-specificity to the different model parameters in an effort to improve our fit to the observed patters of isolation-by-distance. We first used sex-specific dispersal kernels when simulating dispersal events back in time. This model (M1) only slightly improved the proportion of the variance explained ($R^2 = 0.64$, 0.92, 0 for male-male, male-female, and female-female pairs, respectively; Table 1).

However, an important consequence of the strongly female-biased dispersal in the Florida Scrub-Jays is that mean distances between pairs of a given pedigree relationship depend on the sexes of the individuals (Fig 3A, S5 Table). This means that $P_p(d)$, $P_f(d)$, and $P_h(d)$ all vary with the type of comparison (*e.g.*, the proportion of parent-offspring pairs at larger distance bins is higher for female-female pairs than for male-male pairs). Denoting the sexes of the two individuals in a pair as $s_{k,1}$ and $s_{k,2}$, we can rewrite the expected proportion of the genome shared identical-by-descent as (Model 2):

$$\hat{Z} = \sum_{g=1}^{10} \left[\prod_{k=1}^{g-1} (1-M)^2 \left[1 - P_p(d_k, s_{k,1}, s_{k,2}) - P_f(d_k, s_{k,1}, s_{k,2}) - P_h(d_k, s_{k,1}, s_{k,2})\right] \right] \times \\ [P_p(d_g, s_{g,1}, s_{g,2})\mathbb{E}(Z_p) + P_f(d_g, s_{g,1}, s_{g,2})\mathbb{E}(Z_f) + P_h(d_g, s_{g,1}, s_{g,2})\mathbb{E}(Z_h) \\ + 2M(1-M)\mathbb{E}(Z_r) + M^2\mathbb{E}(Z_m)] \quad (5)$$

Running this model with sex-specific estimates of the probabilities ($P_p(d)$, $P_f(d)$, and $P_h(d)$) of being closely related (M2) explained 31% more of the variance in identity-by-descent across distance in male-male comparisons and 84% more of the variance in female-female comparisons (S10 Fig, Table 1). Another consequence of female-biased dispersal is that immigrant individuals from other populations are more likely to be female [3, 4] Indeed, we find that the proportion of female breeders who are immigrants (0.345) is much higher than the proportion of male breeders who are immigrants (0.197). Using sex-specific values for M, the expected proportion of the genome shared identical-by-descent between two individuals is now (Model 3):

$$\hat{Z} = \sum_{g=1}^{10} \left[\prod_{k=1}^{g-1} (1-M(s_{k,1}))(1-M(s_{k,2})) \left[1 - P_p(d_k, s_{k,1}, s_{k,2}) - P_f(d_k, s_{k,1}, s_{k,2}) - P_h(d_k, s_{k,1}, s_{k,2})\right] \right] \times \\ [P_p(d_g, s_{g,1}, s_{g,2})\mathbb{E}(Z_p) + P_f(d_g, s_{g,1}, s_{g,2})\mathbb{E}(Z_f) + P_h(d_g, s_{g,1}, s_{g,2})\mathbb{E}(Z_h) \\ + M(s_{g,1})(1-M(s_{g,2}))\mathbb{E}(Z_r) + (1-M(s_{g,1}))M(s_{g,2})\mathbb{E}(Z_r) + M(s_{g,1})M(s_{g,2})\mathbb{E}(Z_m)] \quad (6)$$

The model with sex-specific immigration parameters (M3) explained a greater proportion of the variance in identity-by-descent across geographic distance for female-female pairs (S10 Fig, Table 1). While incorporating sex-specific parameters improved our ability to

reconstruct the observed pattern of isolation-by-distance, our model overestimated identity-by-descent at longer distances for male-male pairs, and, to a lesser extent, male-female pairs (S10 Fig). We noticed that there is a pattern of isolation-by-distance in the proportion of the genome shared identical-by-descent between immigrant-resident and immigrant-immigrant pairs. Therefore, we allow Z_r and Z_m to vary with distance in our final model, and estimated these parameters in bins of geographic distance (Model 4):

$$\hat{Z} = \sum_{g=1}^{10} \left[\prod_{k=1}^{g-1} (1 - M(s_{k,1}))(1 - M(s_{k,2})) \left[1 - P_p(d_k, s_{k,1}, s_{k,2}) - P_f(d_k, s_{k,1}, s_{k,2}) - P_h(d_k, s_{k,1}, s_{k,2})\right] \right] \times \\ [P_p(d_g, s_{g,1}, s_{g,2})\mathbb{E}(Z_p) + P_f(d_g, s_{g,1}, s_{g,2})\mathbb{E}(Z_f) + P_h(d_g, s_{g,1}, s_{g,2})\mathbb{E}(Z_h) \\ + M(s_{g,1})(1 - M(s_{g,2}))\mathbb{E}(Z_r(d_g)) + (1 - M(s_{g,1}))M(s_{g,2})\mathbb{E}(Z_r(d_g)) + M(s_{g,1})M(s_{g,2})\mathbb{E}(Z_m(d_g))] \tag{7}$$

Under the full model (M4), we no longer see over-inflation of identity-by-descent at longer distances. Identity-by-descent decays fastest with distance in male-male comparisons, which explains why we observe the greatest increase in model fit for male-male pairs.

We repeated these simulations for Z-linked SNPs and obtained similar results, although the R^2 values are consistently smaller (S11 Fig, Table 1). It is not surprising that the Z-linked simulations explain a lower proportion of the variance in mean identity-by-descent with geographic distance than the autosomal simulations, as our estimates of identity-by-descent for the Z chromosome are less accurate. In the Z-linked simulations, we see the greatest improvement in fit once we allow Z_r and Zm to vary with distance (R^2 increases by > 0.2 for all comparisons; Table 1). The importance of Z_r and Z_m in explaining patterns of isolation-by-distance on the Z suggests that a large proportion of our pairs end up having at least one immigrant lineage, which is consistent with the relatively high immigration rate we observe in the field.

References

[1] Wakeley J, King L, Low BS, Ramachandran S. Gene genealogies within a fixed pedigree, and the robustness of Kingman's coalescent. Genetics. 2012;190(4):1433–1445.

[2] Malécot G. Génétique des populations diploïdes naturelles dans le cas d'un seul locus. III. Parenté, mutations et migration. Ann Genet Sel Anim. 1973;5(3):333–361.

[3] Chen N, Cosgrove EJ, Bowman R, Fitzpatrick JW, Clark AG. Genomic Consequences of Population Decline in the Endangered Florida Scrub-Jay. Curr Biol. 2016;26(21):2974–2979.

[4] Fitzpatrick JW, Woolfenden GE, Bowman R. Dispersal distance and its demographic consequences in the Florida Scrub-jay. In: Adams NJ, Slotow RH, editors. Proceedings of the XXII International Ornithological Congress. Durban: South Africa; 1999.
