# Supplementary figures and images for: Deconstructing isolation-by-distance: The genomic consequences of limited dispersal
Source: PLoS Genet. 2017 Aug 3;13(8):e1006911. doi: 10.1371/journal.pgen.1006911 (PMC5542401; doi:10.1371/journal.pgen.1006911)

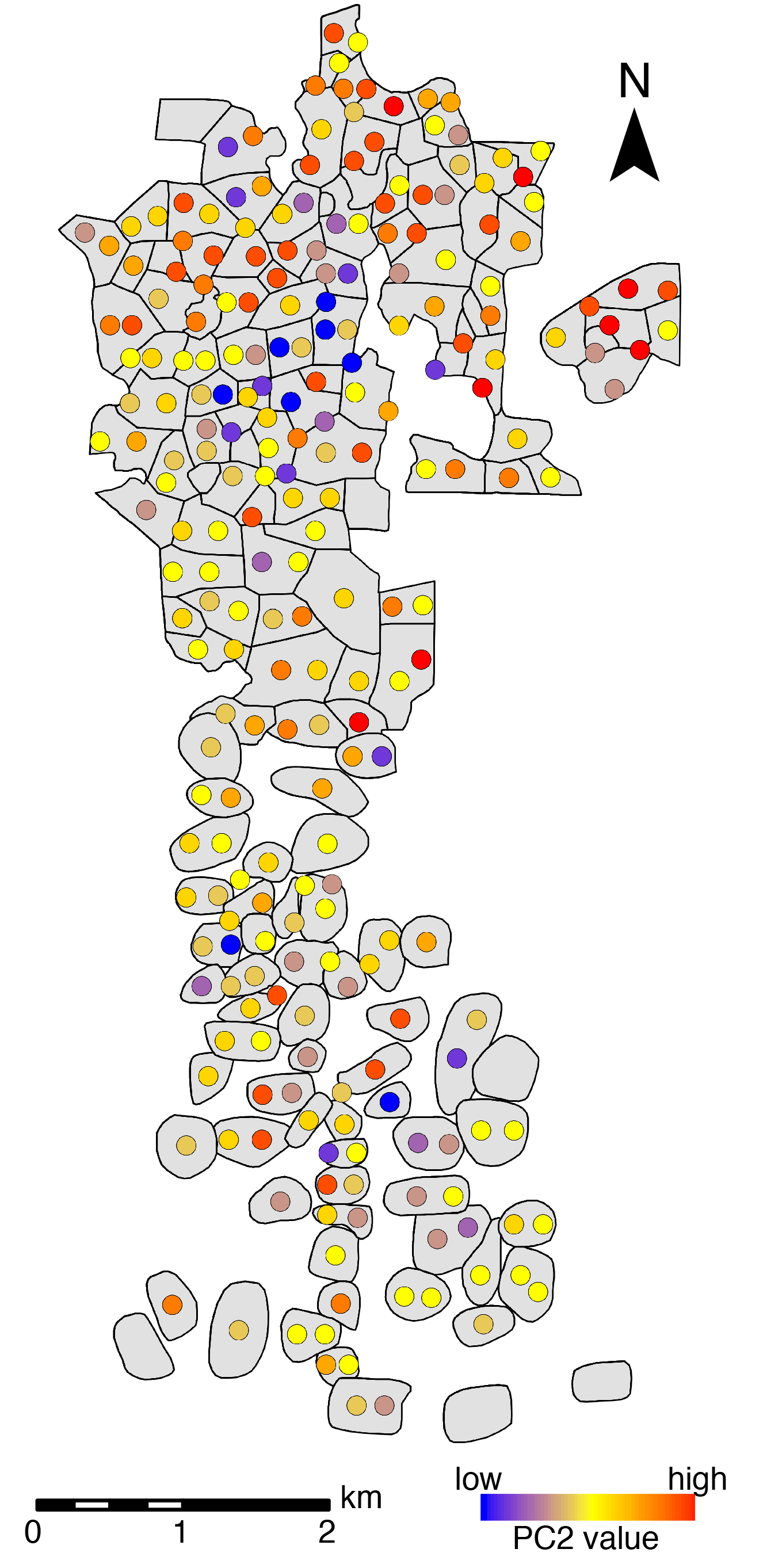

Supplement: S1 Fig — Map of breeding territories (gray polygons) for a representative year (2008) within Archbold Biological Station with individual breeders colored by PC2 values. (TIF) [file pgen.1006911.s009.tif]

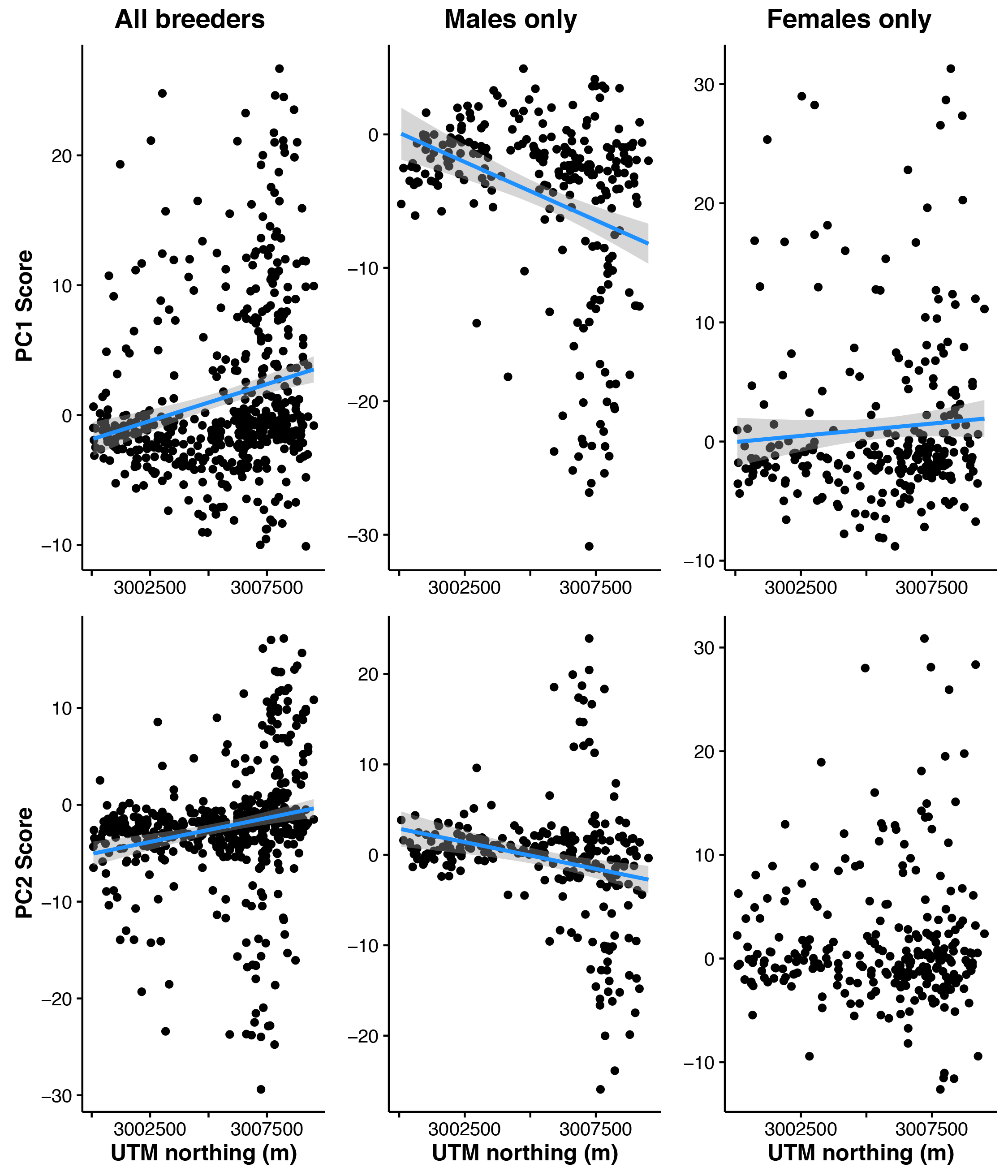

Supplement: S2 Fig — PC score for PCAs conducted on the autosomal SNPs versus UTM northing of the territory centroid for all breeders, males only, and females only. Significant comparisons are shown with a blue linear regression line. See S1 Table for full results. (TIF) [file pgen.1006911.s010.tif]

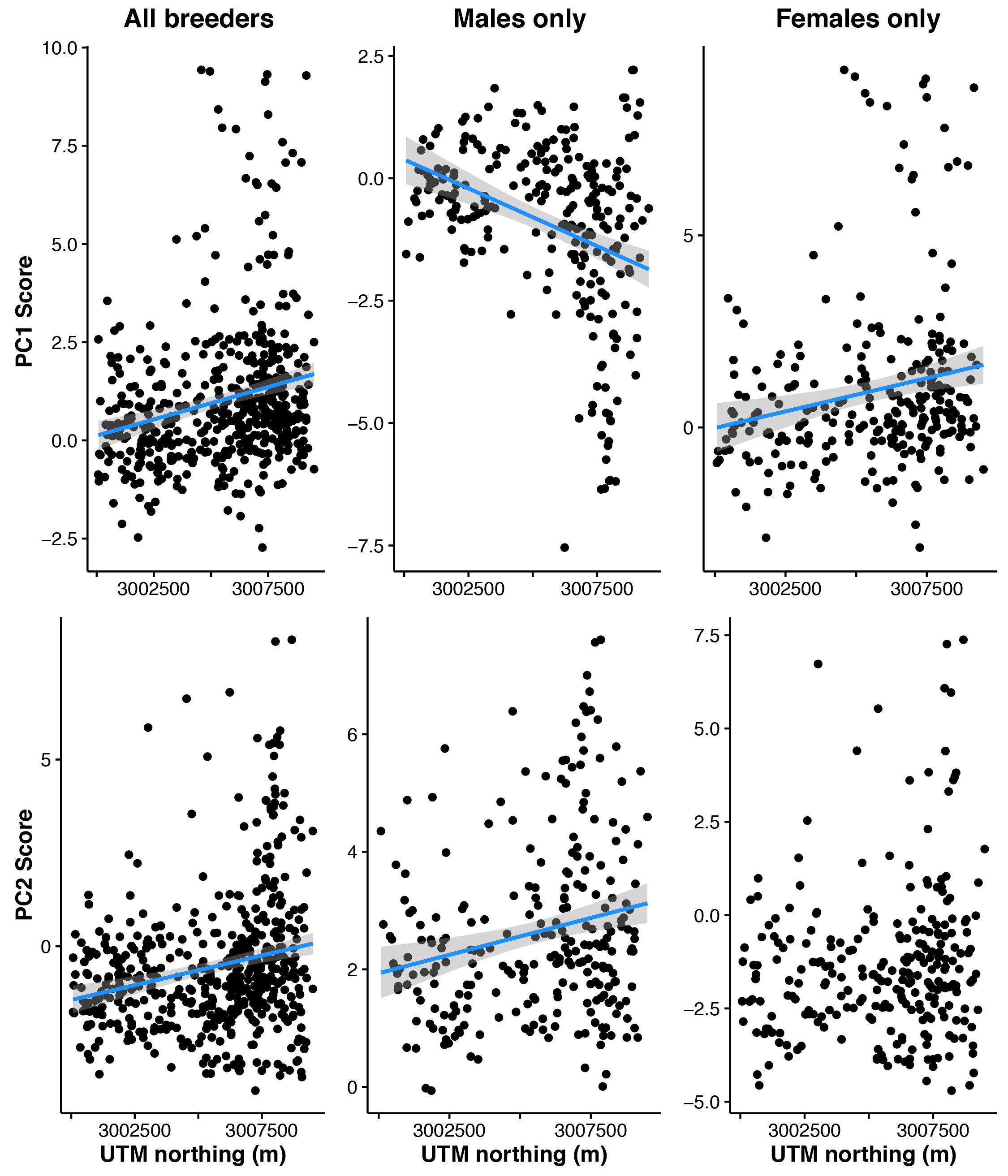

Supplement: S3 Fig — PC score for PCAs conducted on the Z-linked SNPs versus UTM northing of the territory centroid for all breeders, males only, and females only. Significant comparisons are shown with a blue linear regression line. See S1 Table for full results. (TIF) [file pgen.1006911.s011.tif]

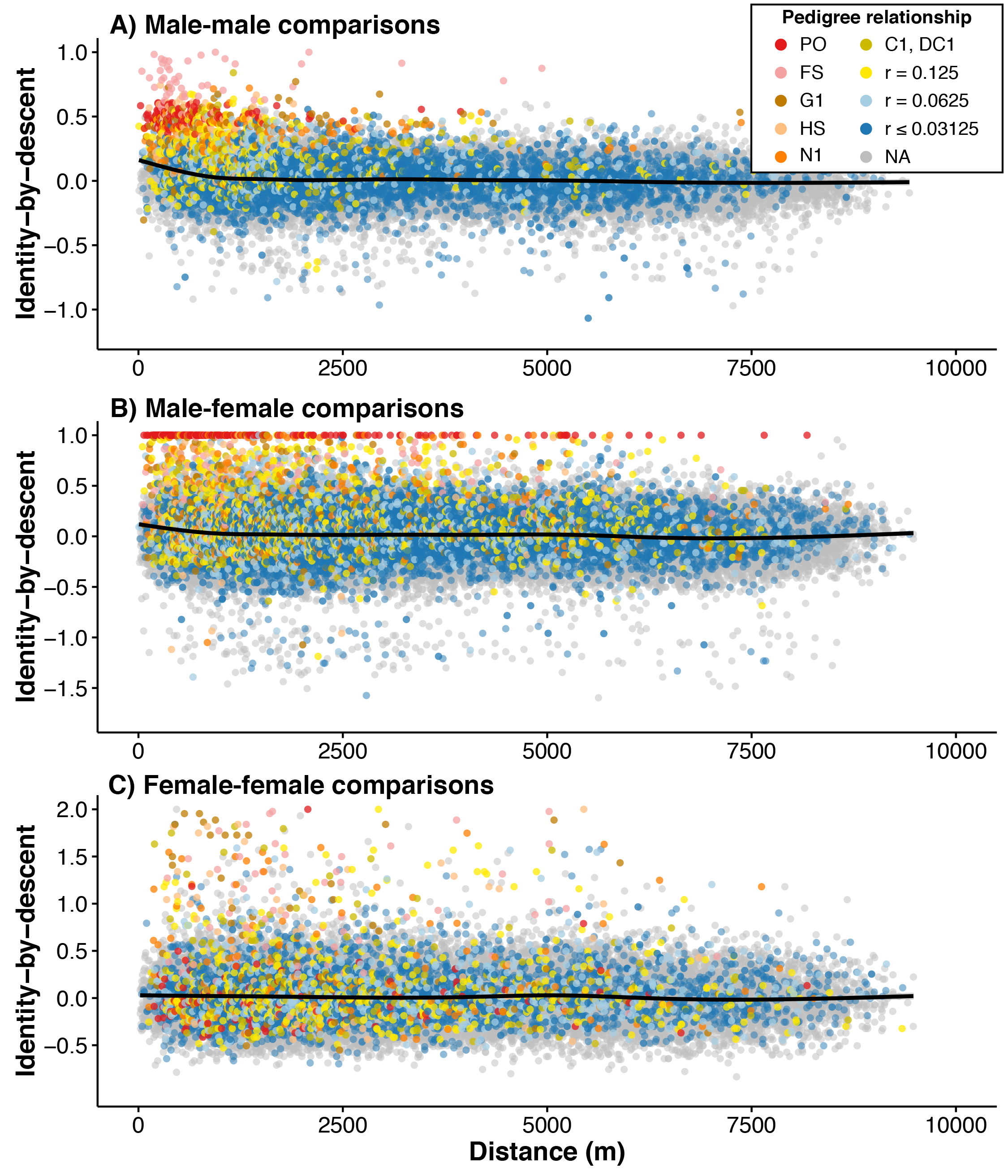

Supplement: S4 Fig — Results are shown for all possible (A) male-male, (B) male-female, and (C) female-female comparisons. Loess curves are shown in each panel. Isolation-by-distance patterns are significantly stronger in male-male (A) comparisons than in either male-female (B) or female-female (C) comparisons. Points are colored by specific pedigree relationship or grouped into a single coefficient of relationship class. Gray points indicate no known pedigree relationship. Note that the scale of the y-axis differs between panels. Pedigree relationship abbreviations: PO = parent-offspring, FS = full-siblings, G1 = grandparent-grandchild, HS = half-siblings, N1 = aunt/uncle-nibling, C1 = first cousins, DC1 = double first cousins. (“Nibling” is a gender-neutral term for niece and nephew). (TIF) [file pgen.1006911.s012.tif]

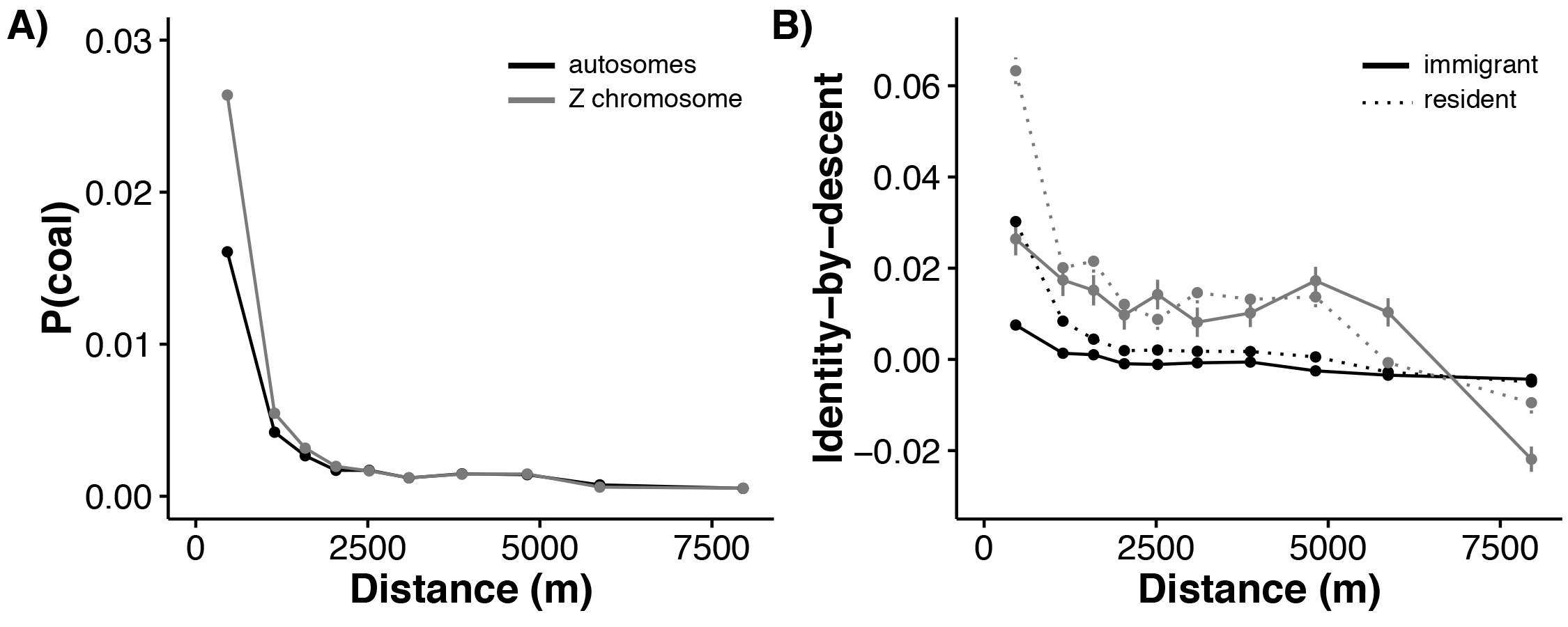

Supplement: S5 Fig — (A) The probability two randomly sampled alleles coalesce in the previous generation for autosomal (black) and Z-linked (gray) markers at different distance bins estimated directly from the population pedigree. The probability two Z-linked alleles coalesce in the previous generation is higher than the probability two autosomal alleles coalesce only at short distances. (B) Unbiased identity-by-descent for autosomal (black) and Z-linked (gray) SNPs versus geographic distance for all possible unique pairwise comparisons, separated into immigrant-immigrant and immigrant-resident pairs (solid line) versus resident-resident pairs (dotted line). Pairs containing an immigrant have higher observed levels of identity-by-descent for Z-linked markers compared to autosomal markers. Here we use untransformed estimates of identity-by-descent to avoid biases introduced by the different numbers of autosomal and Z-linked SNPs. Identity-by-descent values are binned across 10 distance quantiles and shown as mean ± SE. See S2 Text for details. (TIF) [file pgen.1006911.s013.tif]

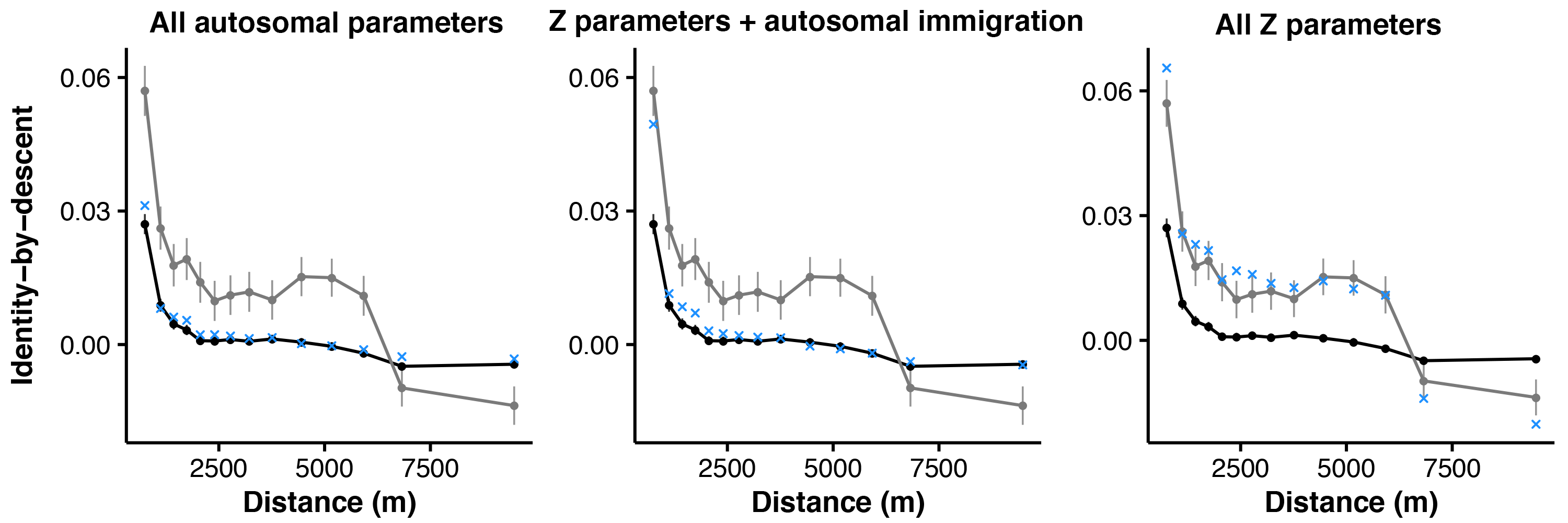

Supplement: S6 Fig — Simulated (blue crosses) and observed (circles and lines) isolation-by-distance patterns for autosomal (black) and Z-linked (gray) markers. Results are shown for three models: the full autosomal model (left), a model with all Z parameters except for identity-by-descent values for immigrants (middle), and the full Z model (right). Substituting in Z values for immigrant identity-by-descent greatly improves the fit of the model, suggesting that the observed pattern of isolation-by-distance on the Z is largely driven by immigration. Untransformed identity-by-descent values are binned across 10 distance quantiles and shown as mean ± SE. See S2 and S3 Text for details. (TIF) [file pgen.1006911.s014.tif]

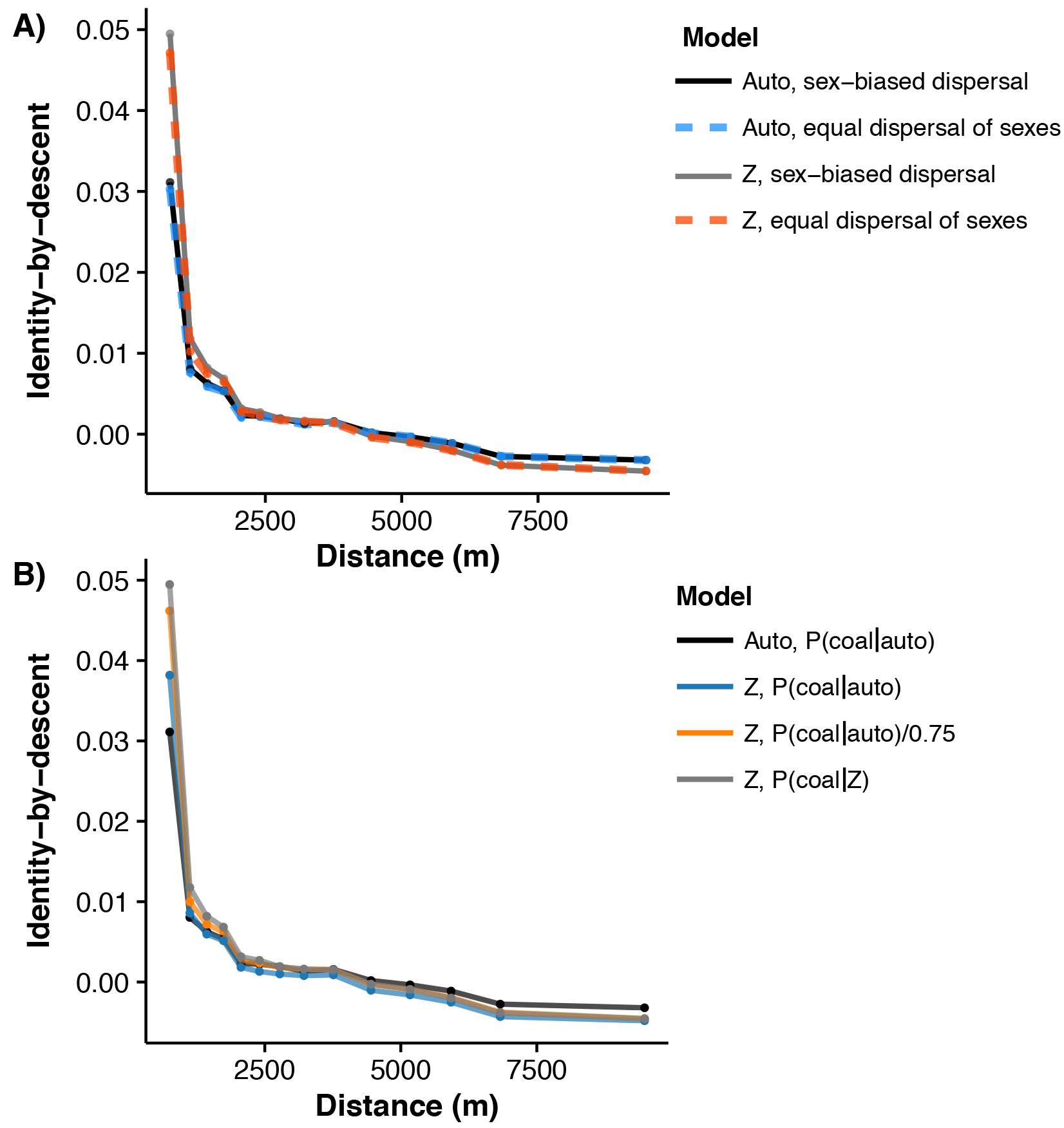

Supplement: S7 Fig — (A) Simulated isolation-by-distance patterns for autosomal (black and blue) and Z-linked (gray and salmon) markers under two different dispersal scenarios. Simulations with sex-biased dispersal are shown as solid lines and simulations with equal dispersal between sexes are shown as dashed lines. Female-biased dispersal slightly elevates predicted identity-by-descent at short distance bins for both types of markers, with a larger effect for Z-linked markers. (B) Simulated isolation-by-distance patterns for four different models: the full autosomal model (black), a model with Z parameters but autosomal coalescent probabilities (blue), a model with Z parameters but autosomal coalescent probabilities divided by 0.75 to simulate the smaller effective population size of the Z (orange), and the full Z-linked model (gray). Both the smaller effective population size of the Z and sex-biased dispersal contribute to higher identity-by-descent for Z-linked markers compared to autosomal markers at short distances. We use autosomal identity-by-descent values for immigrants in all simulations here to generate predictions for autosomal and Z differences without immigration. See S2 Text for details. (TIF) [file pgen.1006911.s015.tif]

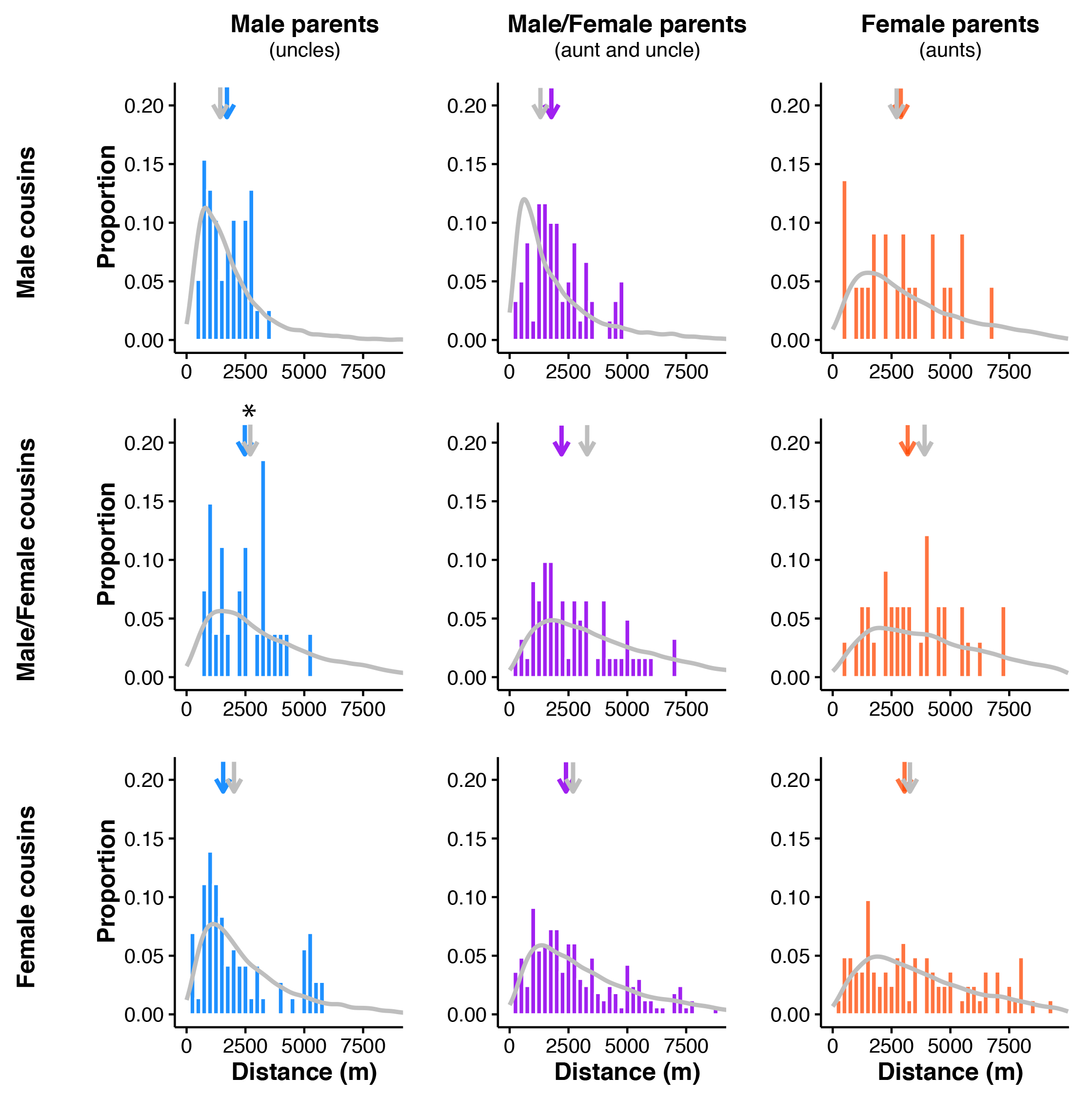

Supplement: S8 Fig — Simulated (gray line) and observed (colored histograms) dispersal values for sex-specific first cousin dispersal simulations. Median values for the simulated (gray) and observed (colored) distributions are indicated by arrows above each plot. Simulated distributions that were significantly different from the observed distribution using the Kolmogorov-Smirnov Test are marked with asterisks above the median arrows. (TIF) [file pgen.1006911.s016.tif]

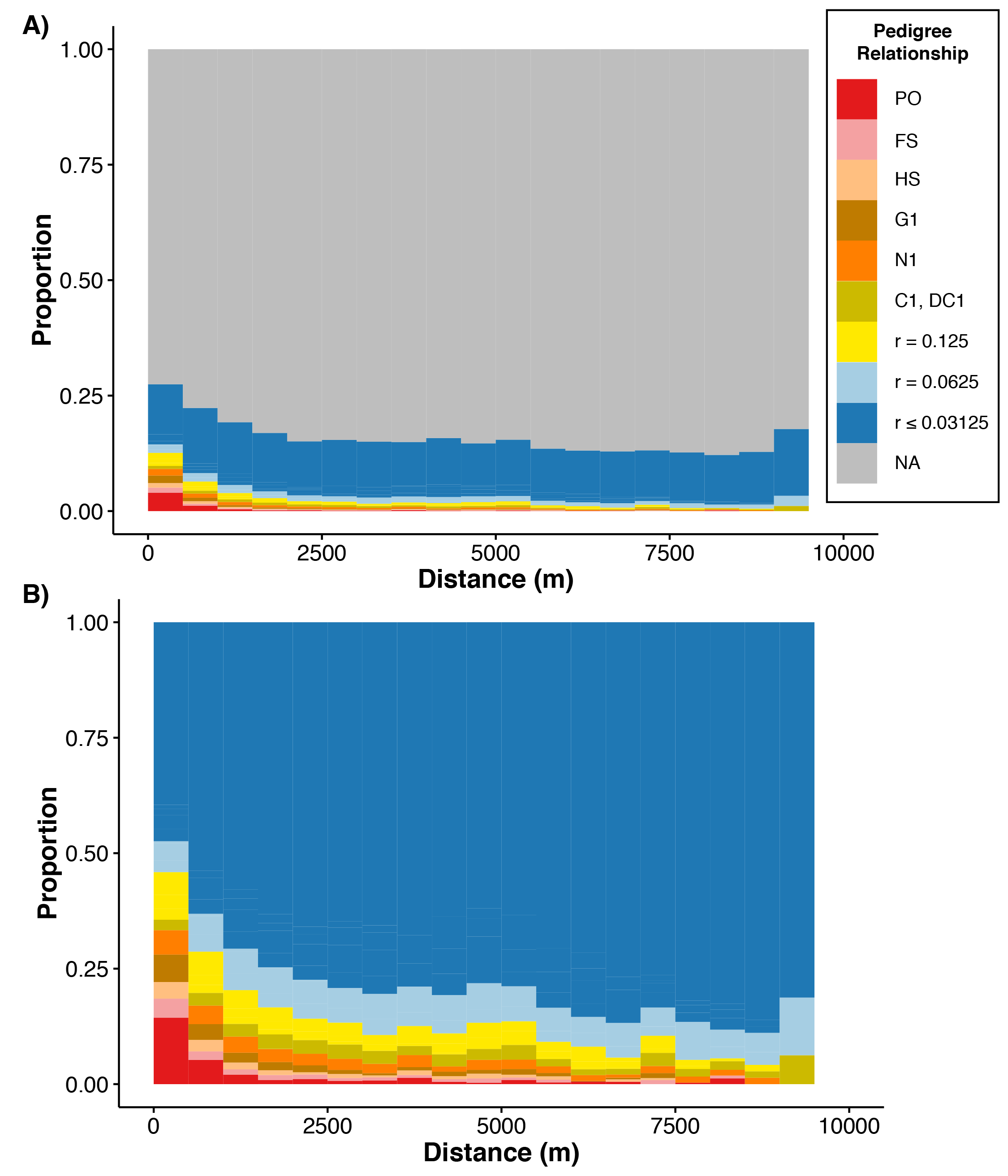

Supplement: S9 Fig — Proportion of various pedigree relationship classes within 500 m distance bins for all unique individual comparisons with (A) including individuals unrelated in the pedigree (NA, gray bars) and (B) excluding individuals unrelated in the pedigree. Colors indicate pedigree relationship and match those in Fig 2 and S4 Fig. Pedigree relationship abbreviations: PO = parent-offspring, FS = full-siblings, G1 = grandparent-grandchild, HS = half-siblings, N1 = aunt/uncle-nibling, C1 = first cousins, DC1 = double first cousins. (“Nibling” is a gender-neutral term for niece and nephew). (TIF) [file pgen.1006911.s017.tif]

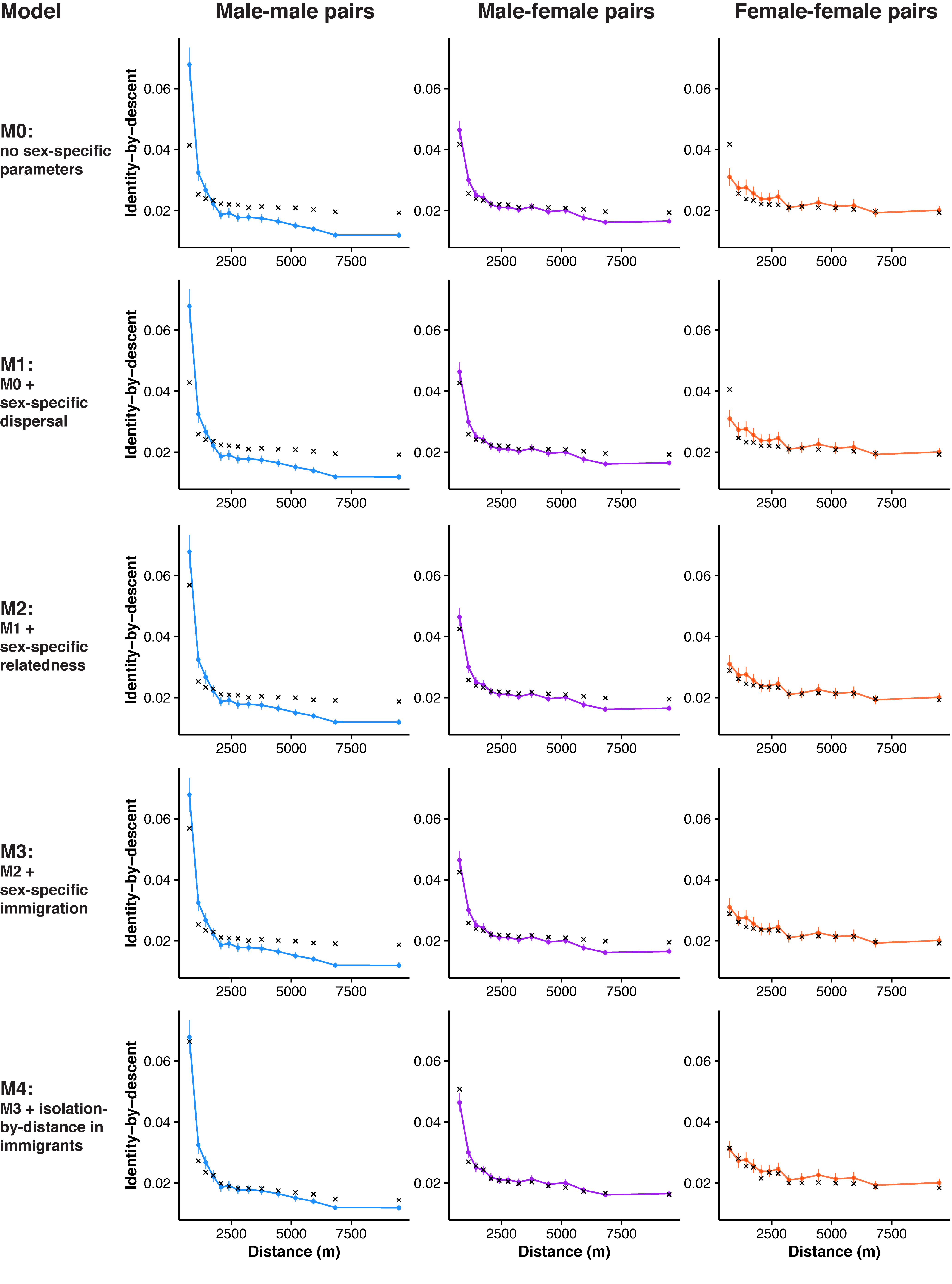

Supplement: S10 Fig — Simulated (black crosses) and observed (colored circles and line) identity-by-descent values are indicated for male-male (blue), male-female (purple), and female-female comparisons (salmon). The coefficient of determination for each model can be found in Table 1. See S3 Text for details. (TIF) [file pgen.1006911.s018.tif]

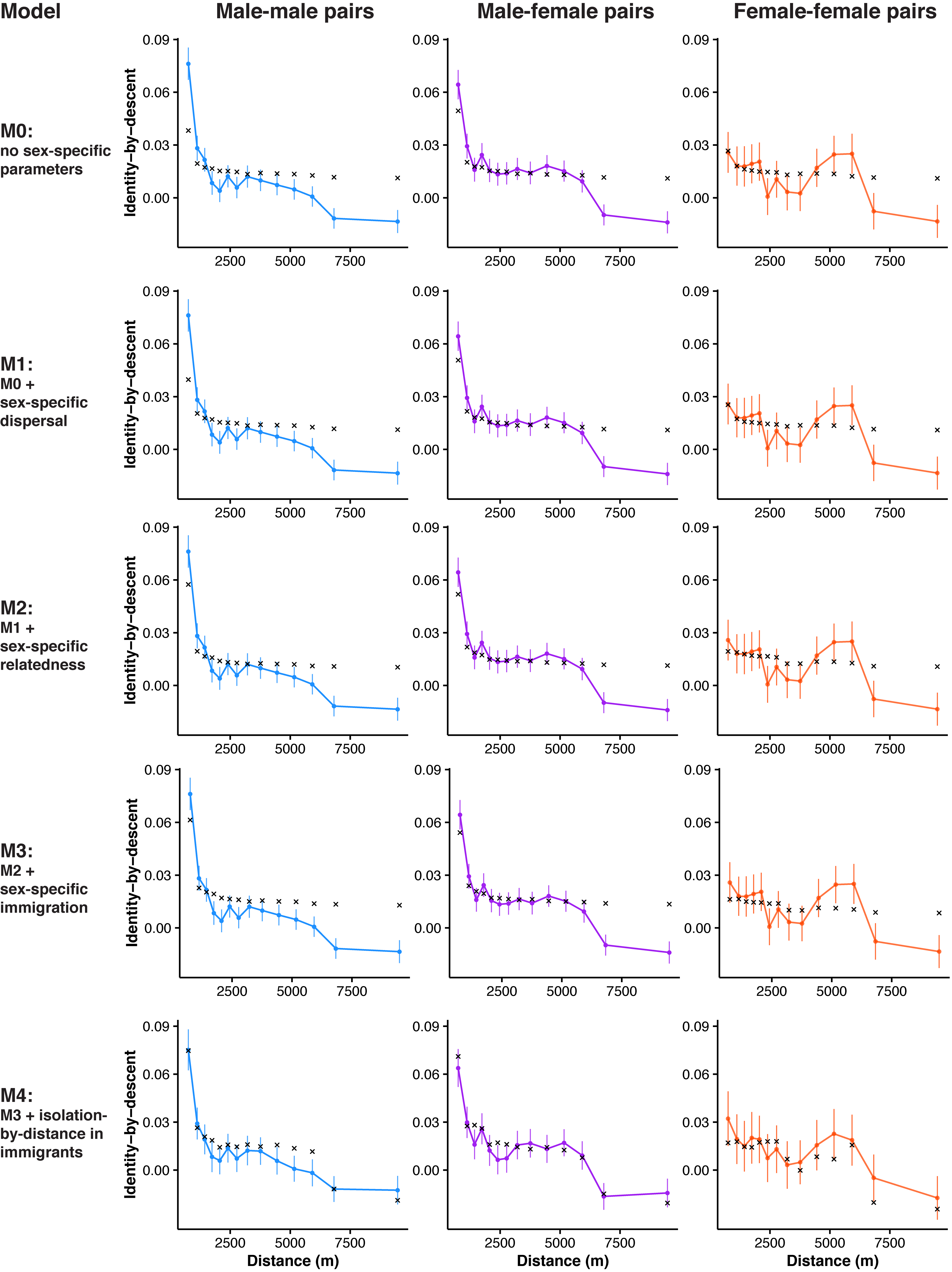

Supplement: S11 Fig — Simulated (black crosses) and observed (colored circles and line) identity-by-descent values are indicated for male-male (blue), male-female (purple), and female-female comparisons (salmon). The coefficient of determination for each model can be found in Table 1. See S3 Text for details. (TIF) [file pgen.1006911.s019.tif]

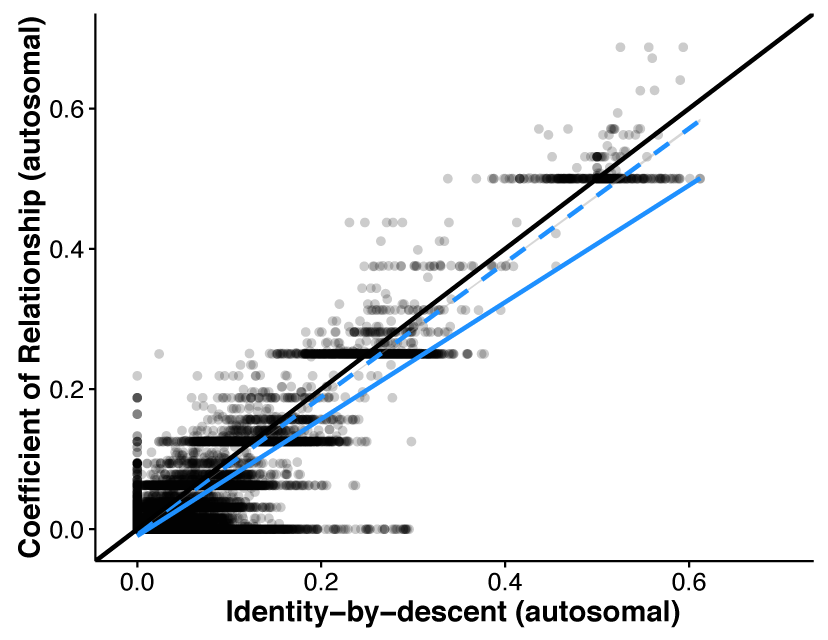

Supplement: S12 Fig — Black line indicates a 1:1 relationship, solid blue line shows the linear regression of all of the data, and the dashed blue line shows the linear regression with r = 0 values removed. (TIF) [file pgen.1006911.s020.tif]

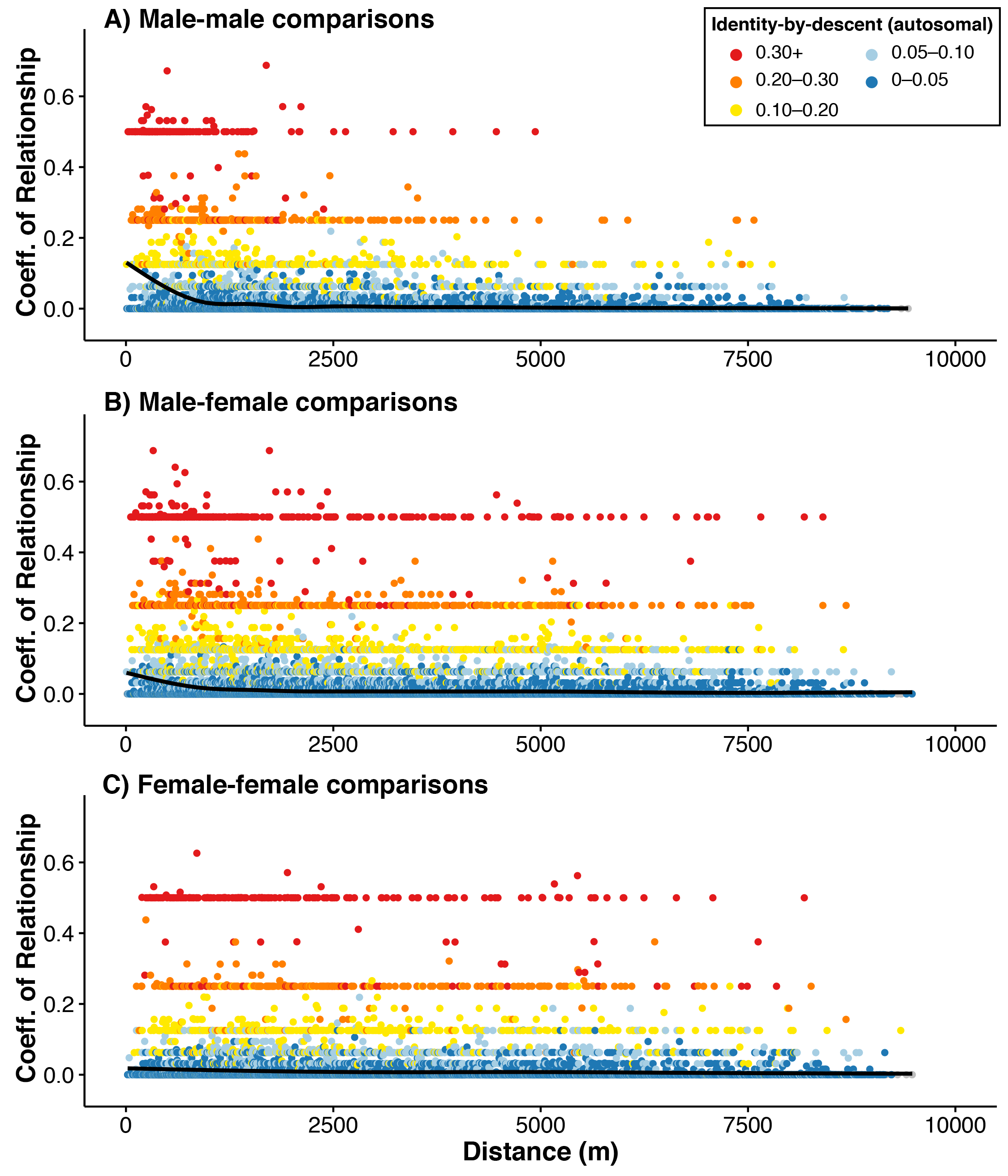

Supplement: S13 Fig — Results are shown for all possible (A) male-male, (B) male-female, and (C) female-female comparisons. Loess curves are shown in each panel. Points are grouped by color into autosomal identity-by-descent classes. Gray points indicate no known pedigree relationship. (TIF) [file pgen.1006911.s021.tif]

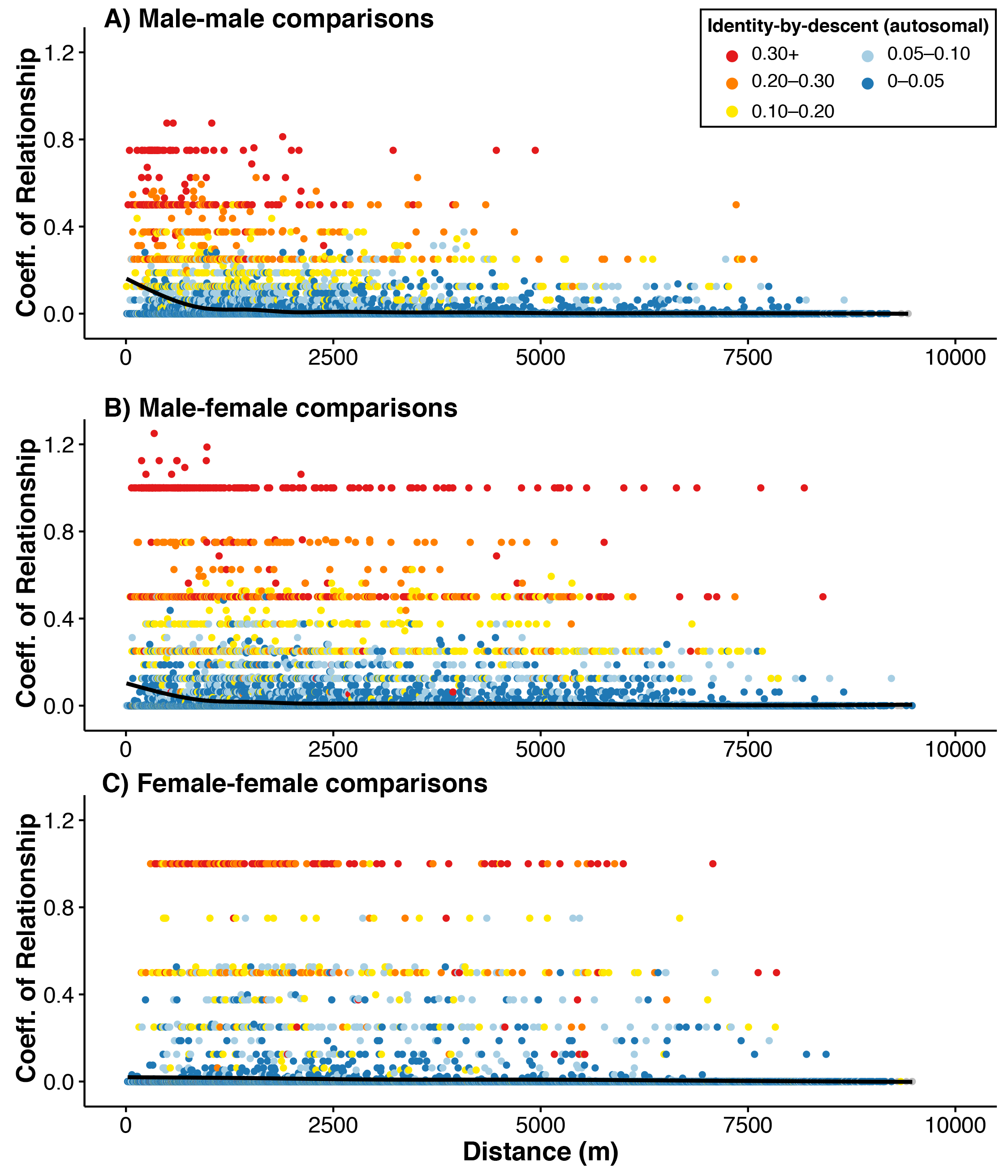

Supplement: S14 Fig — Results are shown for all possible (A) male-male, (B) male-female, and (C) female-female comparisons. Loess curves are shown in each panel. Points are grouped by color into autosomal identity-by-descent classes. Gray points indicate no known pedigree relationship. (TIF) [file pgen.1006911.s022.tif]
