# Supplementary material for: Deconstructing isolation-by-distance: The genomic consequences of limited dispersal
Source: PLoS Genet. 2017 Aug 3;13(8):e1006911. doi: 10.1371/journal.pgen.1006911 (PMC5542401; doi:10.1371/journal.pgen.1006911)
Supplement: S1 Table — Spearman rank correlations comparing the first two PC axes and the UTM northing value of the territory centroid. Each cell contains the Spearman’s ρ value followed by the p-value in parentheses. Significant tests are shown in bold. (DOCX) [file pgen.1006911.s023.docx]

**S1 Table. Correlations between PC axes and UTM northing.** Spearman rank correlations comparing the first two PC axes and the UTM northing value of the territory centroid. Each cell contains the Spearman’s ρ value followed by the *p*-value in parentheses. Significant tests are shown in bold.

|  | Autosomal identity-by-descent | | | Z-linked identity-by-descent | | |
| --- | --- | --- | --- | --- | --- | --- |
|  | All | Male | Female | All | Male | Female |
| PC1 | **0.2730 (<0.0001)** | **-0.3157**  **(<0.0001)** | **0.1546 (0.0124)** | **0.2578 (<0.0001)** | **-0.3416 (<0.0001)** | **0.2242 (0.0003)** |
| PC2 | **0.3213 (<0.0001)** | **-0.3026 (<0.0001)** | -0.0076 (0.9032) | **0.2115 (<0.0001)** | **0.1684 (0.0075)** | 0.1189 (0.0550) |
