# Supplementary material for: Deconstructing isolation-by-distance: The genomic consequences of limited dispersal
Source: PLoS Genet. 2017 Aug 3;13(8):e1006911. doi: 10.1371/journal.pgen.1006911 (PMC5542401; doi:10.1371/journal.pgen.1006911)
Supplement: S2 Table — Mantel correlogram results for all unique individual comparisons of all breeders, male breeders only, and female breeders only across all years. Each cell contains the Mantel correlation coefficient followed by the corrected p-value in parentheses. Significant tests are shown in bold. (DOCX) [file pgen.1006911.s024.docx]

**S2 Table. Mantel correlogram results.** Mantel correlogram results for all unique individual comparisons of all breeders, male breeders only, and female breeders only across all years. Each cell contains the Mantel correlation coefficient followed by the corrected *p*-value in parentheses. Significant tests are shown in bold.

| Distance Midpoint | Autosomal identity-by-descent | | | Z-linked identity-by-descent | | | Autosomal coefficient of relationship | | | Z-linked coefficient of relationship | | |
| --- | --- | --- | --- | --- | --- | --- | --- | --- | --- | --- | --- | --- |
|  | All | Male | Female | All | Male | Female | All | Male | Female | All | Male | Female |
| 500 | **-0.1141­**  **(0.0002)** | **-0.2048­**  **(0.0002)** | **-0.0402­**  **(0.0002)** | **-0.0479­**  **(0.0002)** | **-0.0972­**  **(0.0002)** | **-0.0155­**  **(0.0218)** | **-0.1222­**  **(0.0002)** | **-0.2207­**  **(0.0002)** | **-0.0432­**  **(0.0002)** | **-0.1072­**  **(0.0002)** | **-0.211­0**  **(0.0002)** | **-0.0256­**  **(0.0004)** |
| 1000 | **-0.0385­**  **(0.0003)** | **-0.0595­**  **(0.0003)** | **-0.0215­**  **(0.0014)** | **-0.0162­**  **(0.0015)** | **-0.0331­**  **(0.0003)** | -0.0106­  (0.1712) | **-0.0419­**  **(0.0003)** | **-0.0625­**  **(0.0003)** | **-0.0225­**  **(0.0006)** | **-0.0405­**  **(0.0003)** | **-0.0722­**  **(0.0003)** | **-0.0243­**  **(0.0006)** |
| 1500 | **-0.0134­**  **(0.0004)** | **-0.011­0**  **(0.0409)** | **-0.0216­**  **(0.0021)** | -0.0064­  (0.1134) | -0.0038­  (0.3262) | -0.0068­  (0.3788) | **-0.0121­**  **(0.0005)** | -0.0077­  (0.0941) | **-0.0215­**  **(0.0009)** | **-0.0154­**  **(0.0004)** | **-0.0151­**  **(0.0069)** | **-0.0246­**  **(0.0004)** |
| 2000 | **0.0082­**  **(0.0070** | **0.0196­**  **(0.0006)** | 0.0014­  (0.4178) | 0.002­0  (0.3285) | 0.0088­  (0.2820) | -0.0012­  (0.8133) | **0.0145­**  **(0.0005)** | **0.0284­**  **(0.0005)** | 0.0018­  (0.3857) | **0.0092­**  **(0.0014)** | **0.0261­**  **(0.0005)** | -0.0099­  (0.0836) |
| 2500 | **0.0114­**  **(0.0020)** | **0.0259­**  **(0.0006)** | -0.008­0  (0.1976) | 0.0053­  (0.3402) | 0.0107­  (0.3045) | 0.0045­  (0.8108) | **0.0172­**  **(0.0006)** | **0.0298­**  **(0.0006)** | 0.0013­  (0.7713) | **0.0148­**  **(0.0006)** | **0.0283­**  **(0.0006)** | 0.003­  (0.4366) |
| 3000 | **0.0171­**  **(0.0007)** | **0.0225­**  **(0.0009)** | 0.0145­  (0.0548) | 0.0027­  (0.6391) | 0.0003­  (0.6523) | 0.0079­  (0.8469) | **0.0209­**  **(0.0007)** | **0.0278­**  **(0.0007)** | **0.0164­**  **(0.0100)** | **0.0175­**  **(0.0007)** | **0.0294­**  **(0.0007)** | 0.0086­  (0.2310) |
| 3500 | **0.0133­**  **(0.0015)** | **0.0243­**  **(0.0008)** | 0.0134­  (0.0832) | 0.005­0  (0.6278) | 0.0041­  (0.9695) | 0.0099­  (0.6749) | **0.0165­**  **(0.0008)** | **0.0295­**  **(0.0008)** | **0.0145­**  **(0.0264)** | **0.0136­**  **(0.0008)** | **0.0289­**  **(0.0008)** | 0.0099­  (0.2090) |
| 4000 | **0.0134­**  **(0.0009)** | **0.0247­**  **(0.0009)** | 0.0023­  (0.7117) | -0.0011­  (0.9587) | 0.0022  ­(1.0000) | -0.0003­  (1.0000) | **0.015­0**  **(0.0009)** | **0.0278­**  **(0.0009)** | 0.0016­  (1.0000) | **0.0152­**  **(0.0009)** | **0.0261­**  **(0.0009)** | 0.0075­  (0.3080) |
| 4500 | **0.0197­**  **(0.0010)** | **0.031­0**  **(0.0010)** | 0.0121­  (0.1248) | 0.0007­  (1.0000) | 0.0114­  (0.5004) | -0.007­0  (1.0000) | **0.0171­**  **(0.0010)** | **0.0295­**  **(0.0010)** | 0.0062­  (0.5507) | **0.0162­**  **(0.0010)** | **0.0336­**  **(0.0010)** | 0.0039­  (0.6548) |
| 5000 | **0.0144­**  **(0.0011)** | **0.0321­**  **(0.0011)** | 0.0079­  (0.3896) | -0.0044­  (1.0000) | 0.0146­  (0.3216) | **-0.0186­**  **(0.0484)** | **0.0131­**  **(0.0011)** | **0.0366­**  **(0.0011)** | 0.0030  ­(1.0000) | **0.0146­**  **(0.0011)** | **0.0382­**  **(0.0011)** | 0.0071­  (0.4655) |
