# Supplementary material for: Deconstructing isolation-by-distance: The genomic consequences of limited dispersal
Source: PLoS Genet. 2017 Aug 3;13(8):e1006911. doi: 10.1371/journal.pgen.1006911 (PMC5542401; doi:10.1371/journal.pgen.1006911)
Supplement: S3 Table — Counts of different known relationship pairs in the full pedigree of the Archbold population of Florida Scrub-Jays. The expected coefficient of relationship for each pair of relatives is also included. Note that the actual pedigree-based coefficient of relationship for a given pair may be higher than the expected value due to inbreeding in the population. Pedigree relationship abbreviations: PO = parent-offspring, FS = full-siblings, HS = half-siblings, G1 = grandparent-grandchild, N1 = aunt/uncle-nibling, DC1 = double first cousins, C1 = first cousins, G2 = great-grandparent-great-grandchild, GN = great-aunt/uncle-grand-nibling, N2 = half-aunt/uncle-half-nibling, C0.5 = half-cousins, C1r1 = first cousins once removed, G3 = great-great-grandparent-great-great-grandchild, C2 = second cousins, C2r1 = second cousins once removed, C3 = third cousins, C4 = fourth cousins. (DOCX) [file pgen.1006911.s025.docx]

**S3 Table. Counts of known relationship pairs in the pedigree.** Counts of different known relationship pairs in the full pedigree of the Archbold population of Florida Scrub-Jays. The expected coefficient of relationship for each pair of relatives is also included. Note that the actual pedigree-based coefficient of relationship for a given pair may be higher than the expected value due to inbreeding in the population. Pedigree relationship abbreviations: PO = parent-offspring, FS = full-siblings, HS = half-siblings, G1 = grandparent-grandchild, N1 = aunt/uncle-nibling, DC1 = double first cousins, C1 = first cousins, G2 = great-grandparent-great-grandchild, GN = great-aunt/uncle-grand-nibling, N2 = half-aunt/uncle-half-nibling, C0.5 = half-cousins, C1r1 = first cousins once removed, G3 = great-great-grandparent-great-great-grandchild, C2 = second cousins, C2r1 = second cousins once removed, C3 = third cousins, C4 = fourth cousins.

| Pedigree relationship | # of pairs in pedigree | Expected coefficient of relationship |
| --- | --- | --- |
| PO | 19346 | 0.5 |
| FS | 39704 | 0.5 |
| HS | 95168 | 0.25 |
| G1 | 19744 | 0.25 |
| N1 | 83442 | 0.25 |
| DC1 | 354 | 0.25 |
| C1 | 71797 | 0.125 |
| G2 | 19892 | 0.125 |
| GN | 81498 | 0.125 |
| N2 | 96604 | 0.125 |
| C0.5 | 139129 | 0.0625 |
| C1r1 | 144148 | 0.0625 |
| G3 | 19106 | 0.0625 |
| C2 | 94869 | 0.03125 |
| C2r1 | 169164 | 0.015625 |
| C3 | 93114 | 0.0078125 |
| C4 | 60065 | 0.001953125 |
