# Supplementary material for: Deconstructing isolation-by-distance: The genomic consequences of limited dispersal
Source: PLoS Genet. 2017 Aug 3;13(8):e1006911. doi: 10.1371/journal.pgen.1006911 (PMC5542401; doi:10.1371/journal.pgen.1006911)
Supplement: S4 Table — Wilcoxon rank sum test results comparing the distribution of distances between pairs in different pedigree relationship classes. Sequential comparisons were performed between pairs with pedigree coefficient of relationship (r) and pairs with half that relationship (0.5r). Significant tests are shown in bold. (DOCX) [file pgen.1006911.s026.docx]

**S4 Table. Distribution of distances between pairs of varying pedigree relationship.** Wilcoxon rank sum test results comparing the distribution of distances between pairs in different pedigree relationship classes. Sequential comparisons were performed between pairs with pedigree coefficient of relationship (*r*) and pairs with half that relationship (0.5*r*). Significant tests are shown in bold.

| Pedigree coefficient of relationship | | Wilcoxon rank sum test | |
| --- | --- | --- | --- |
| *r* | 0.5*r* | *U* | *p*-value |
| **0.5** | **0.25** | **211475.5** | **< 2.2x10^-16^** |
| **0.25** | **0.125** | **500201.5** | **5.27x10^-11^** |
| **0.125** | **0.0625** | **927983** | **2.20x10^-5^** |
| 0.0625 | 0.03125 | 1257984 | 0.11 |
| **0.03125** | **0.015625** | **1015128** | **0.00045** |
| **0.015625** | **0.0078125** | **730483.5** | **0.0032** |
