# Supplementary material for: Deconstructing isolation-by-distance: The genomic consequences of limited dispersal
Source: PLoS Genet. 2017 Aug 3;13(8):e1006911. doi: 10.1371/journal.pgen.1006911 (PMC5542401; doi:10.1371/journal.pgen.1006911)
Supplement: S5 Table — Wilcoxon rank sum test results comparing the distribution of distances between all individuals within specific pedigree relationship classes (as shown in boxplots in Fig 3A). All possible pairwise tests were conducted on distances between male-male, male-female, and female-female comparisons. Significant tests are shown in bold. Pedigree relationship abbreviations: PO = parent-offspring, FS = full-siblings, G1 = grandparent-grandchild, N1 = aunt/uncle-nibling, C1 = first cousins, C2 = second cousins, C3 = third cousins, NA = no known pedigree relationship. MM = male-male pairs, MF = male-female pairs, FF = female-female pairs. (DOCX) [file pgen.1006911.s027.docx]

**S5 Table. Distribution of distances between sex-specific pairs with a given pedigree relationship.** Wilcoxon rank sum test results comparing the distribution of distances between all individuals within specific pedigree relationship classes (as shown in boxplots in Fig 3A). All possible pairwise tests were conducted on distances between male-male, male-female, and female-female comparisons. Significant tests are shown in bold. Pedigree relationship abbreviations: PO = parent-offspring, FS = full-siblings, G1 = grandparent-grandchild, N1 = aunt/uncle-nibling, C1 = first cousins, C2 = second cousins, C3 = third cousins, NA = no known pedigree relationship. MM = male-male pairs, MF = male-female pairs, FF = female-female pairs.

| Pedigree relationship | MM-FF | | MM-MF | | MF-FF | |
| --- | --- | --- | --- | --- | --- | --- |
|  | *U* | *p*-value | *U* | *p*-value | *U* | *p*-value |
| PO | **2349.5** | **<0.0001** | **10664** | **<0.0001** | **8161** | **<0.0001** |
| FS | **417** | **<0.0001** | **1432** | **<0.0001** | **3009** | **0.0351** |
| G1 | **1963** | **<0.0001** | **6361** | **<0.0001** | **6211.5** | **0.0089** |
| N1 | **5723** | **<0.0001** | **14494** | **<0.0001** | **17798** | **0.0148** |
| C1 | **5976.5** | **<0.0001** | **16643** | **0.0049** | **15660** | **0.0003** |
| C2 | **16028** | **<0.0001** | **28790** | **0.0102** | **30804** | **0.0431** |
| C3 | **9456** | **0.0099** | **24174** | **0.0347** | 16301 | 0.2757 |
| NA | **380460000** | **<0.0001** | **713610000** | **0.0002** | 795300000 | 0.1007 |
