# Supplementary material for: Deconstructing isolation-by-distance: The genomic consequences of limited dispersal
Source: PLoS Genet. 2017 Aug 3;13(8):e1006911. doi: 10.1371/journal.pgen.1006911 (PMC5542401; doi:10.1371/journal.pgen.1006911)
Supplement: S6 Table — Tests of autosomal isolation-by-distance (measured as the difference between identity-by-descent (IBD) at distance bin 0 and the overall mean IBD) and the strength of isolation-by-distance (measured by δ) for different subsets of the data. Mean values are listed with 95% CI in parentheses. (DOCX) [file pgen.1006911.s028.docx]

**S6 Table. Isolation-by-distance in different subsets of the data.** Tests of autosomal isolation-by-distance (measured as the difference between identity-by-descent (IBD) at distance bin 0 and the overall mean IBD) and the strength of isolation-by-distance (measured by δ) for different subsets of the data. Mean values are listed with 95% CI in parentheses.

| Dataset | IBD_0_-meanIBD | δ |
| --- | --- | --- |
| all | 0.036 (0.033, 0.039) | 644.77  (632.13, 657.83) |
| *r* < 0.5 | 0.015 (0.014, 0.016) | 741.36  (708.07, 776.48) |
| *r* < 0.25 | 0.006 (0.005, 0.007) | 870.10  (793.13, 990.68) |
| *r* < 0.125 | 0.003 (0.002, 0.004) | 1089.93  (872.18, 1590.54) |
| *r* < 0.0625 | 0.001 (0.001, 0.002) | 1665.66  (968.27, 3204.35) |
