# Supplementary material for: Deconstructing isolation-by-distance: The genomic consequences of limited dispersal
Source: PLoS Genet. 2017 Aug 3;13(8):e1006911. doi: 10.1371/journal.pgen.1006911 (PMC5542401; doi:10.1371/journal.pgen.1006911)
Supplement: S7 Table — Kolmogorov-Smirnov (KS) and Wilcoxon rank sum (WRS) test results comparing simulated and observed distributions and medians, respectively, between individuals within specific pedigree relationship classes (as shown in Fig 6). Each cell contains the test statistic followed by the p-value in parentheses with significance based on a Bonferroni-corrected threshold of p < 0.004. Significant tests are shown in bold. Pedigree relationship abbreviations: FS = full-siblings, N1 = aunt/uncle-nibling, C1 = first cousins, C2 = second cousins. (DOCX) [file pgen.1006911.s029.docx]

**S7 Table.** **Results from dispersal simulations for different related pairs.** Kolmogorov-Smirnov (KS) and Wilcoxon rank sum (WRS) test results comparing simulated and observed distributions and means, respectively, between individuals within specific pedigree relationship classes (as shown in Fig 6). Each cell contains the test statistic followed by the *p*-value in parentheses with significance based on a Bonferroni-corrected threshold of *p* < 0.004. Significant tests are shown in bold. Pedigree relationship abbreviations: FS = full-siblings, N1 = aunt/uncle-nibling, C1 = first cousins, C2 = second cousins.

| Pedigree relationship | Male-male comparisons | | Male-female comparisons | | Female-female comparisons | |
| --- | --- | --- | --- | --- | --- | --- |
|  | KS Test | WRS Test | KS Test | WRS Test | KS Test | WRS Test |
| FS | 0.3049 (0.0093) | 189620 (0.0042) | 0.0615 (0.9603) | 328450 (0.6288) | 0.2369 (0.0164) | 250000 (0.0651) |
| N1 | **0.3439 (<0.0001)** | **313990 (0.0002)** | 0.1207 (0.0923) | 1171100 (0.0624) | 0.1398 (0.2642) | 302890 (0.0399) |
| C1 | 0.1037 (0.3315) | 436200 (0.5419) | **0.1603 (0.0002)** | **2128600 (<0.0001)** | 0.1345 (0.1455) | 389100 (0.3303) |
| C2 | 0.1361 (0.053) | **579890 (0.0033)** | 0.1278 (0.0155) | **1763400 (0.0002)** | **0.19966 (<0.0001)** | **774160 (<0.0001)** |
