# Supplementary material for: Deconstructing isolation-by-distance: The genomic consequences of limited dispersal
Source: PLoS Genet. 2017 Aug 3;13(8):e1006911. doi: 10.1371/journal.pgen.1006911 (PMC5542401; doi:10.1371/journal.pgen.1006911)
Supplement: S8 Table — Kolmogorov-Smirnov (KS) and Wilcoxon rank sum (WRS) test results comparing simulated and observed distributions and medians, respectively, between sex-specific first cousin comparisons (as shown in S8 Fig). Each cell contains the test statistic followed by the p-value in parentheses with significance based on a Bonferroni-corrected threshold of p < 0.0055. Significant tests are shown in bold. MM = male-male pairs, MF = male-female pairs, FF = female-female pairs. (DOCX) [file pgen.1006911.s030.docx]

**S8 Table. Results from sex-specific first cousin dispersal simulations.** Kolmogorov-Smirnov (KS) and Wilcoxon rank sum (WRS) test results comparing simulated and observed distributions and medians, respectively, between sex-specific first cousin comparisons (as shown in S8 Fig). Each cell contains the test statistic followed by the *p*-value in parentheses with significance based on a Bonferroni-corrected threshold of *p* < 0.0055. Significant tests are shown in bold. MM = male-male pairs, MF = male-female pairs, FF = female-female pairs.

|  | Sex of parental individuals | | | | | |
| --- | --- | --- | --- | --- | --- | --- |
|  | MM | | MF | | FF | |
| Sex of cousins | KS Test | WRS Test | KS Test | WRS Test | KS Test | WRS Test |
| MM | 0.1327 (0.5011) | 181140 (0.4428) | 0.1461 (0.0946) | 400660 (0.0981) | 0.2368 (0.0976) | 157750 (0.1301) |
| MF | **0.2636 (0.0005)** | 480680 (0.0077) | 0.0989 (0.0816) | 1794000 (0.0532) | 0.2158 (0.0070) | **759860 (0.0009)** |
| FF | 0.1313 (0.8439) | 115560 (0.6817) | 0.0984 (0.4107) | 400660 (0.0981) | 0.2061 (0.1223) | 193630 (0.0963) |
