# Supplementary material for: Deconstructing isolation-by-distance: The genomic consequences of limited dispersal
Source: PLoS Genet. 2017 Aug 3;13(8):e1006911. doi: 10.1371/journal.pgen.1006911 (PMC5542401; doi:10.1371/journal.pgen.1006911)
Supplement: S9 Table — Expected identity-by-descent values are the coefficient of relationship for different pairs. For the expected Z-linked identity-by-descent of male-female parent-offspring pairs, we averaged that of father-daughter and mother-son pairs. For the expected Z-linked identity-by-descent of half-siblings, we averaged that of paternal half-siblings and maternal half-siblings. See [72] for derivation. MM = male-male pairs, MF = male-female pairs, FF = female-female pairs. (DOCX) [file pgen.1006911.s031.docx]

**S9 Table.** **Expected autosomal and Z-linked identity-by-descent values used in the coalescent simulations.** Expected identity-by-descent values are the coefficient of relationship for different pairs. For the expected Z-linked identity-by-descent of male-female parent-offspring pairs, we averaged that of father-daughter and mother-son pairs. For the expected Z-linked identity-by-descent of half-siblings, we averaged that of paternal half-siblings and maternal half-siblings. See [72] for derivation. MM = male-male pairs, MF = male-female pairs, FF = female-female pairs.

| Pedigree relationship | Autosomal | Z-linked | | |
| --- | --- | --- | --- | --- |
|  |  | MM | MF | FF |
| Parent-offspring | 0.5 | 0.5 | 1 | 0 |
| Full-siblings | 0.5 | 0.75 | 0.5 | 1 |
| Half-siblings | 0.25 | 0.375 | 0.25 | 0.5 |
